# Neuromodulatory effect of 4-(methylthio)butyl isothiocyanate against 3-nitropropionic acid induced oxidative impairments in human dopaminergic SH-SY5Y cells via BDNF/CREB/TrkB pathway

**DOI:** 10.1038/s41598-023-31716-3

**Published:** 2023-03-17

**Authors:** Prabhjot Kaur, Shivani Attri, Davinder Singh, Farhana Rashid, Sharabjit Singh, Avinash Kumar, Harjot Kaur, Neena Bedi, Saroj Arora

**Affiliations:** 1 Department of Botanical and Environmental Sciences, Guru Nanak Dev University, Amritsar, 143005 India; 2 Department of Molecular Genetics, The Ohio State University, Columbus, OH 43210 USA; 3 Department of Biotechnology, Punjabi University, Patiala, 147001 India; 4 Department of Pharmaceutical Sciences, Guru Nanak Dev University, Amritsar, 143005 India

**Keywords:** Cell biology, Drug discovery, Neuroscience, Plant sciences

## Abstract

Mitochondrial impairment, energetic crisis and elevated oxidative stress have been demonstrated to play a pivotal role in the pathological processes of Huntington’s disease (HD). 3-Nitropropionic acid (3-NPA) is a natural neurotoxin that mimics the neurological dysfunctions, mitochondrial impairments and oxidative imbalance of HD. The current investigation was undertaken to demonstrate the neuroprotective effect of 4-(methylthio)butyl isothiocyanate (4-MTBITC) against the 3-NPA induced neurotoxicity in human dopaminergic SH-SY5Y cells. The experimental evidence of oxidative DNA damage by 3-NPA was elucidated by pBR322 DNA nicking assay. In contrast, the 4-MTBITC considerably attenuated the DNA damage, suggesting its free radical scavenging action against 3-NPA and Fenton's reagent. The dose and time-dependent increase of 3-NPA revealed its neurotoxic dose as 0.5 mM after 24 h of treatment of SH-SY5Y cells in MTT assay. In order to determine the optimal dose at which 4-MTBITC protects cell death, the 3-NPA (IC_50_) induced cells were pretreated with different concentrations of 4-MTBITC for 1 h. The neuroprotective dose of 4-MTBITC against 3-NPA was found to be 0.25 μM. Additionally, the elevated GSH levels in cells treated with 4-MTBITC indicate its propensity to eliminate reactive species generated as a result of 3-NPA-induced mitochondrial dysfunction. Likewise, it was determined through microscopic and flow cytometric experiments that 3-NPA's induced overproduction of reactive species and a decline in mitochondrial membrane potential (MMP) could be efficiently prevented by pre-treating cells with 4-MTBITC. To elucidate the underlying molecular mechanism, the RT-qPCR analysis revealed that the pre-treatment of 4-MTBITC effectively protected neuronal cells against 3-NPA-induced cell death by preventing Caspase-3 activation, Brain-derived neurotrophic factor (BDNF) upregulation, activation of cAMP response element-binding protein (CREB) and Nrf2 induction. Together, our findings lend credence to the idea that pre-treatment with 4-MTBITC reduced 3-NPA-induced neurotoxicity by lowering redox impairment, apoptotic state, and mitochondrial dysfunction. The present work, in conclusion, presented the first proof that the phytoconstituent 4-MTBITC supports the antioxidant system, BDNF/TrkB/CREB signaling, and neuronal survival in dopaminergic SH-SY5Y cells against 3-NPA-induced oxidative deficits.

## Introduction

According to World Health Organization (WHO), the entire global population having age group of 60 years-and-above is being predicted to become double over the next three decades^1^. With increasing global life expectancy rate, the prevalence of heterogeneous group of neurodegenerative diseases (NDDs) such as Alzheimer’s disease, Amyotrophic lateral sclerosis, Huntington’s disease, and Parkinson’s disease is also rising day by day. As a result of these neurological disorders, an estimated range of 6–8 million people die annually^2^. Excessive levels of reactive oxygen species (ROS), neuroinflammation, mitochondrial impairment, and disturbances in protein homeostasis (proteostasis) contribute to the progression of various NDDs. Huntington's disease (HD) is a progressive, terminal, incurable NDD caused by an extended repeat of CAG codon in the Huntingtin gene that encodes an abnormally long sequence of polyglutamine in the Huntingtin protein (Htt)^3,4^. This autosomal dominant disorder is characterized by motor, cognitive, and psychiatric disturbances^5^. Atypical involuntary and voluntary movements linked with corticostriatal neuronal loss are the additional hallmarks^6^. The mutant protein mHtt, according to a number of investigations, may facilitate the breakdown of the mitochondrial electron transport chain. As a result of mitochondrial dysfunction, a predominant increase in the free radical production and oxidative stress occurs, which causes gradual brain damage^7,8^.

Experimental evidences collected in recent years suggest that activation of the BDNF/CREB/TrkB pathway is beneficial in HD^9^. Brain-derived neurotrophic factor (BDNF) is essential for the control of brain functioning. BDNF stimulates tyrosine kinase B (TrkB), which helps neurons differentiation and survival^10^. TrkB causes the upregulation of cAMP response element-binding protein (CREB), a downstream effector, to become active. multiple biological processes, including neuron survival, differentiation, and synaptic transmission in the brain, depend on CREB^11,12^. *β*-Nitropropionic acid (3-nitropropionic acid, 3-NPA, C_3_H_5_NO_4_) is a mitochondrial toxin that produces striatal alterations similar to those observed in the brain of patients suffering from Huntington’s disease. It irreversibly inhibits the complex II of the electron transport chain^13^. 3-NPA down-regulates Glutathione (GSH) and initiate pro-oxidant effects which causes bioenergetics deficits in human cells by affecting mitochondrial function and redox balance^14,15^. Moreover, it reduces the levels of neurotrophic factors such as BDNF which is critical for normal health of brain cells^16^.

Since, the pathophysiology of HD is still unknown and existing treatment options provide symptomatic relief which is not sufficiently feasible to restore the quality of life. In light of this, antioxidants are the most desirable alternative natural multi-target therapeutic candidates with minimum side effects particularly for the brain, a tissue rich in fatty acids and is especially vulnerable to ROS-mediated mitochondrial damage and oxidative stress than other organs^17^. As indicated by Melrose et al*.*^18^, the antioxidant properties of activated glucosinolate compounds are known to guard the brain health. Food rich antioxidants, especially isothiocyanates present in Brassica vegetables, have been reported to display neuroprotective effects in several experimental paradigms^19^.

*Eruca sativa* (Mill) Thell. is an annual plant of the family Brassicaceae commonly known as rogula, salad rocket, or taramira. It is rich in gluco-4-methylthiobutyl isothiocyanate, a glucosinolate which breaks down to form a highly volatile glucosinolate hydrolytic product i.e. 4-(methylthio)butyl isothiocyanate (4-MTBITC) or erucin^20^. 4-MTBITC is a structural analog of sulforaphane (SFN) which known for its neuroprotective potential. A comparative study done by Morroni et al*.*^21^ demonstrates the adaptive neuroprotective benefits of SFN and 4-MTBITC against in vitro and in vivo PD model through induction of GSH via nuclear factor erythroid 2–related factor 2 (Nrf2) activation. In recent experimental study, SFN has been confirmed to exert neuroprotective actions through normalizing mitochondrial function and suppressing oxidative stress via Nrf2/HO-1 pathway in SH-SY5Y cell line^22^. Similar to this, 4-MTBITC can support healthy neuronal function in wide range of animal and human tissues^23^. Contrary to SFN, the biological effects of 4-MTBITC are not well supported by experimental data. 4-MTBITC exhibits a cytoprotective, anti-inflammatory and antioxidant tendency against numerous NDDs^24–26^. Therefore, the current study addresses the ameliorating effect of 4-MTBITC against the modulation of molecular and biochemical markers in neurodegeneration caused by 3-NPA in neuroblastoma (SH-SY5Y) cells.

## Results

### Isolation and characterization of 4-MTBITC

A quick and efficient hydro-distillation method resulted in the extraction of 4-MTBITC from seeds of plant *Eruca sativa* with > 99 percent purity. It was detected by the UHPLC-PDA as a single peak. The m/z ions were also examined and their results were contrasted with published data. The characterization was done with ^1^H NMR (Supplementary Fig. S1).

### 4-MTBITC protects against pBR322 DNA damage

**Figure 1:**
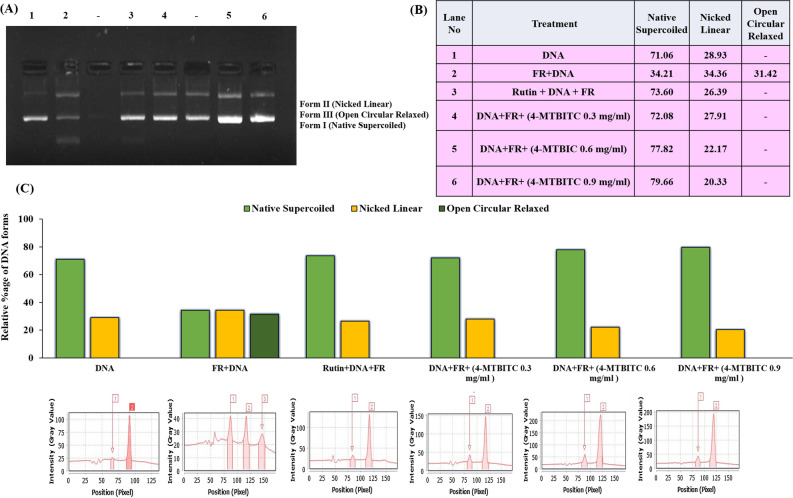
(**A**) Lane 1–6 represent protective effect of 4-MTBITC in pBR322 DNA nicking with Fenton’s reagent (FR). 1: native supercoiled DNA; 2: FR treated DNA; 3: FR treated DNA and rutin (100 μg/ml); 4–6: the FR treated DNA with varying concentrations (0.3, 0.6 and 0.9 mg/ml) of 4-MTBITC; (**B**) Tabular; and (**C**) Graphical representation of quantitative proportion of different DNA plasmid forms after treatment using Lab Image software. All the treatments in each lane of gel were run in parallel. The supplementary file is provided with the raw full-length gel corresponding to figure number used here in the manuscript.

**Figure 2:**
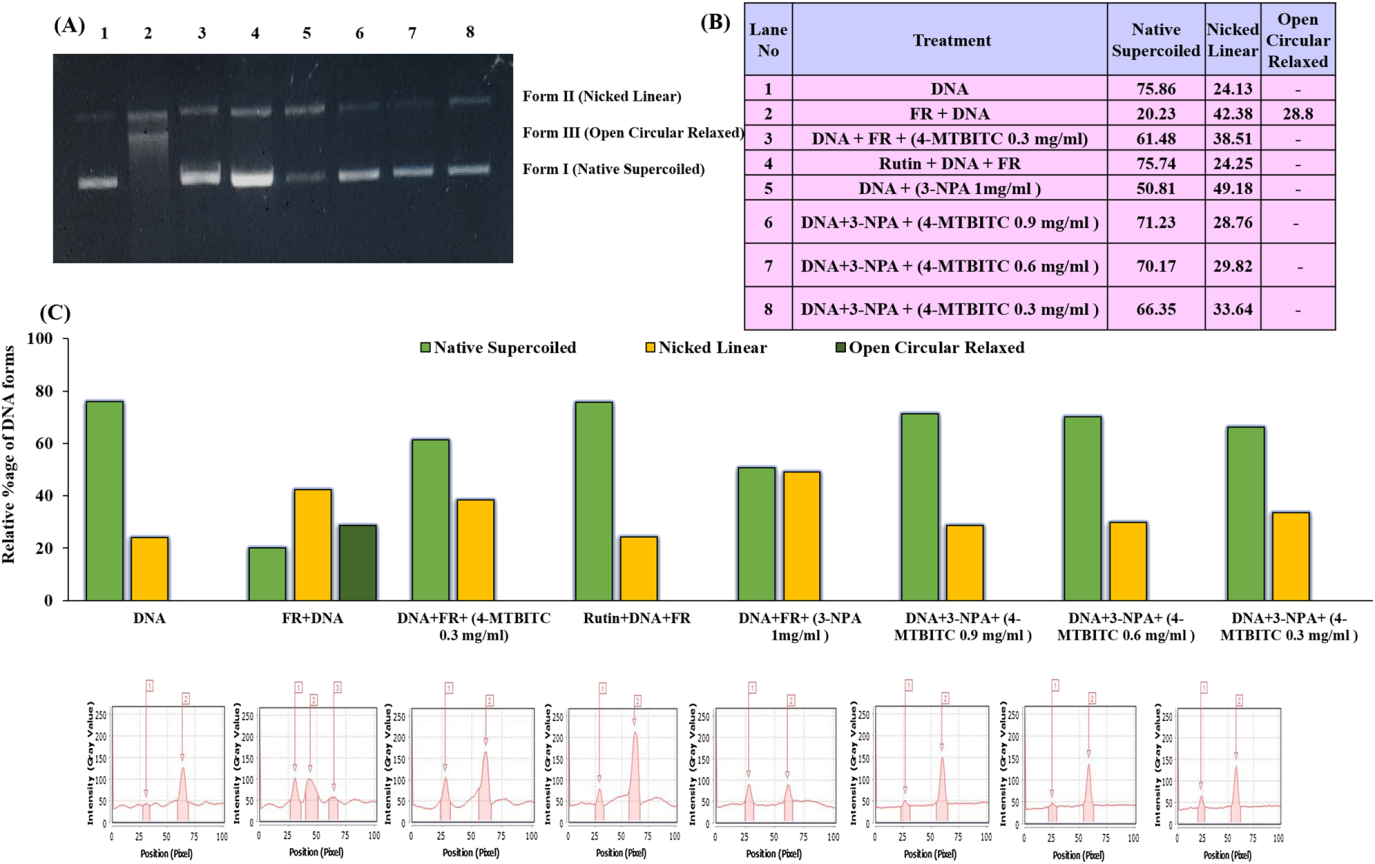
(**A**) Protective effect of 4-MTBITC on pBR322 DNA damage triggered by Fenton’s reagent (FR) and 3-NPA. Lane 1, native supercoiled DNA; Lane 2, FR treated DNA; Lane 3, FR treated DNA + 0.3 mg/ml 4-MTBITC; lane 4, FR treated DNA and rutin (100 μg/ml); lane 5, DNA + 3-NPA (1 mg/ml); lanes 6–8, 3-NPA treated DNA with varying concentrations (0.3, 0.6 and 0.9 mg/ml) of 4-MTBITC; (**B**) Tabular; and (**C**) Graphical representation of quantitative proportion of different DNA plasmid forms after treatment. All the treatments in each lane of gel were run in parallel. The supplementary file is provided with the raw full-length gel corresponding to figure number used here in the manuscript.

DNA Nicking assay is a reliable method to quantify the pBR322 DNA damage with conversion of its native supercoiled form into open circular and nicked linear forms. In the present study, we have analyzed 3-NPA as a nicking agent. 4-MTBITC showed the DNA protective potential against the free radicals generated by Fenton’s reagent and protection increased with increase in concentration of 4-MTBITC from 0.3 mg/ml to 0.9 mg/ml. At highest concentration, 4-MTBITC showed 80% of preserved native supercoiled plasmid DNA (Fig. 1). Similarly, the protective effect of 4-MTBITC was observed against the 3-NPA induced oxidative damage. The intensity of supercoiled form of plasmid was found to be preserved with increase in the concentration of 4-MTBITC against the damage caused by 1 mg/ml concentration of 3-NPA for 1 h. These results are well presented in the band profile of plasmid (pBR322) DNA in Fig. 2.

### SH-SY5Y cell viability detection

#### 3-NPA treatment induced neurotoxicity in SH-SY5Y cells

**Figure 3:**
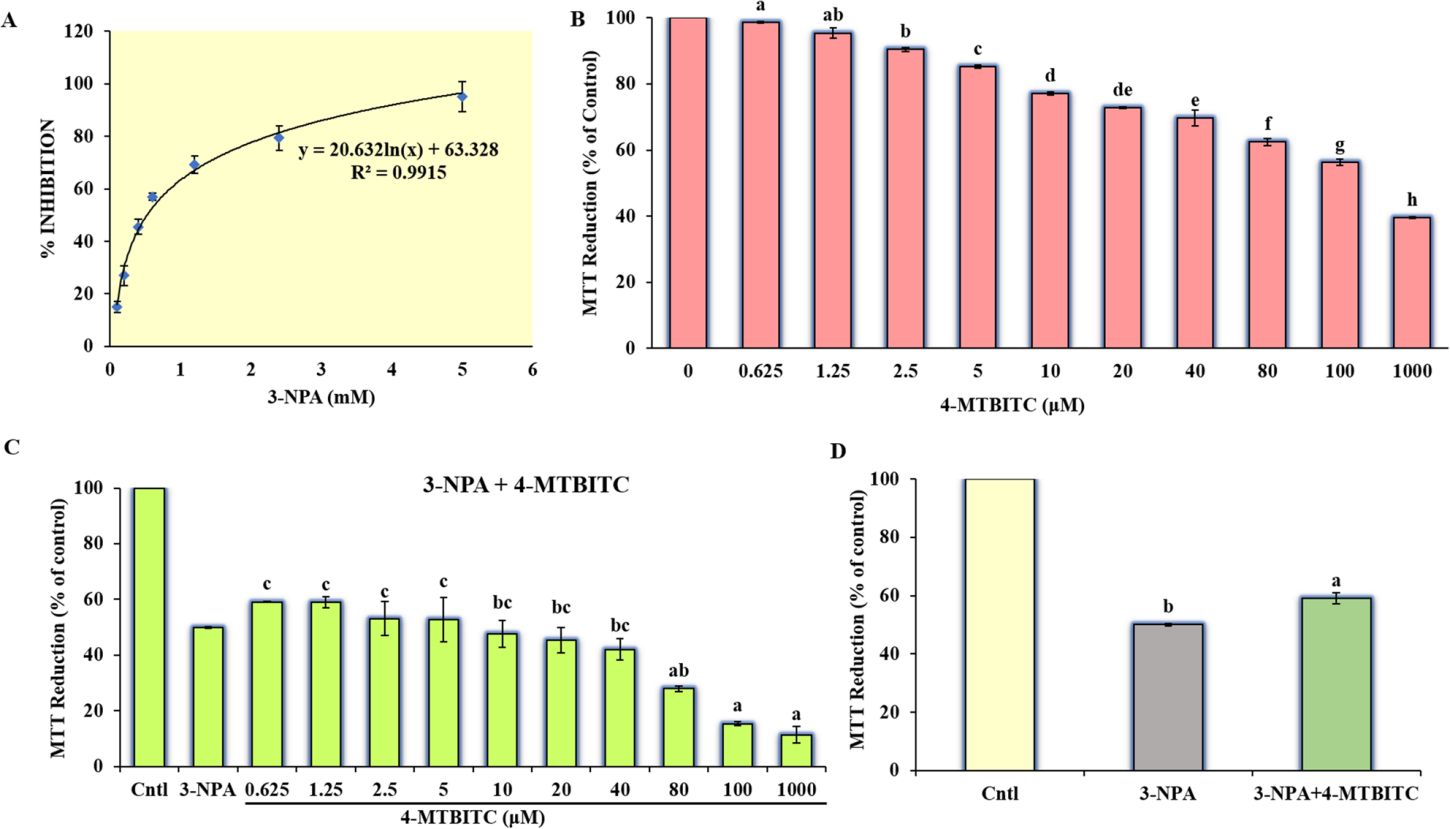
(**A**) Dose-dependent cytotoxicity profile of 3-NPA (0.1–5 mM) in SH-SY5Y cells; (**B**) Dose-dependent cellular viability of 4-MTBITC alone; (**C**) Dose-dependent study of cellular viability of pretreatment of 4-MTBITC (0.625–1000 μM) against 0.5 mM 3-NPA exposed cells for 24 h; (**D**) Representation of cellular viability of optimal cytotoxic dose of 3-NPA (0.5 mM) and optimal cytoprotective dose of 4-MTBITC (1.25 µM) used in combination with 3-NPA. MTT assay results detected the cellular viability as the percentage of reduction in MTT with comparison to control. The measurements are represented as the mean ± SE at the level of significance *p* ≤ 0.05. Small letters as data labels indicate statistical difference comparatively to control by one-way ANOVA, followed by the Tukey post hoc test.

To determine the effect of 3-NPA on viability of the human neuroblastoma (SH-SY5Y), the cells were treated with different concentrations of 3-NPA. Figure 3A shows the concentration–response study of 3-NPA (0.1–15 mM) for 24 h. It exhibited concentration-dependent cell death with IC_50_ of 0.5 mM. As a result, the 0.5 mM dose was selected to induce neurotoxicity in SH-SY5Y cells for further studies.

#### 4-MTBITC attenuated 3-NPA neurotoxicity in neuroblastoma (SH-SY5Y) cells

The neuroprotective potential of 4-MTBITC against 3-NPA toxicity was evaluated by the MTT reduction assay. A 1 h prior exposure of cells with different doses of 4-MTBITC (0.625–1000 μM) against 0.5 mM 3-NPA for 24 h was observed (Fig. 3C). It was found that more than 50% cells were significantly viable at the lower doses (1.25–5 μM) of 4-MTBITC (Fig. 3C). The protective concentration of 4-MTBITC at which roughly 60% of SH-SY5Y cells survived the toxicity generated by 3-NPA was determined to be 1.25 μM. The dose of 1.25 μM was selected throughout the subsequent studies as the protective concentration at which approximately 60% SH-SY5Y cells survived the toxicity induced by 3-NPA. Moreover, the optimal cytotoxic dose of 3-NPA (0.5 mM) and optimal cytoprotective dose of 4-MTBITC (1.25 µM) used in combination with 3-NPA in MTT assay were statistically significant comparatively to control [F-ratio: 22.2801; *p* = 0.0091] (Fig. 3D). However, the results of 4-MTBITC alone exhibited dose-dependent reduction in the metabolic ability of MTT as depicted in the Fig. 3B indicating the cell survival at lower doses only.

### 4-MTBITC increased the levels of intracellular reduced glutathione (GSH) in SH-SY5Y cells

**Figure 4:**
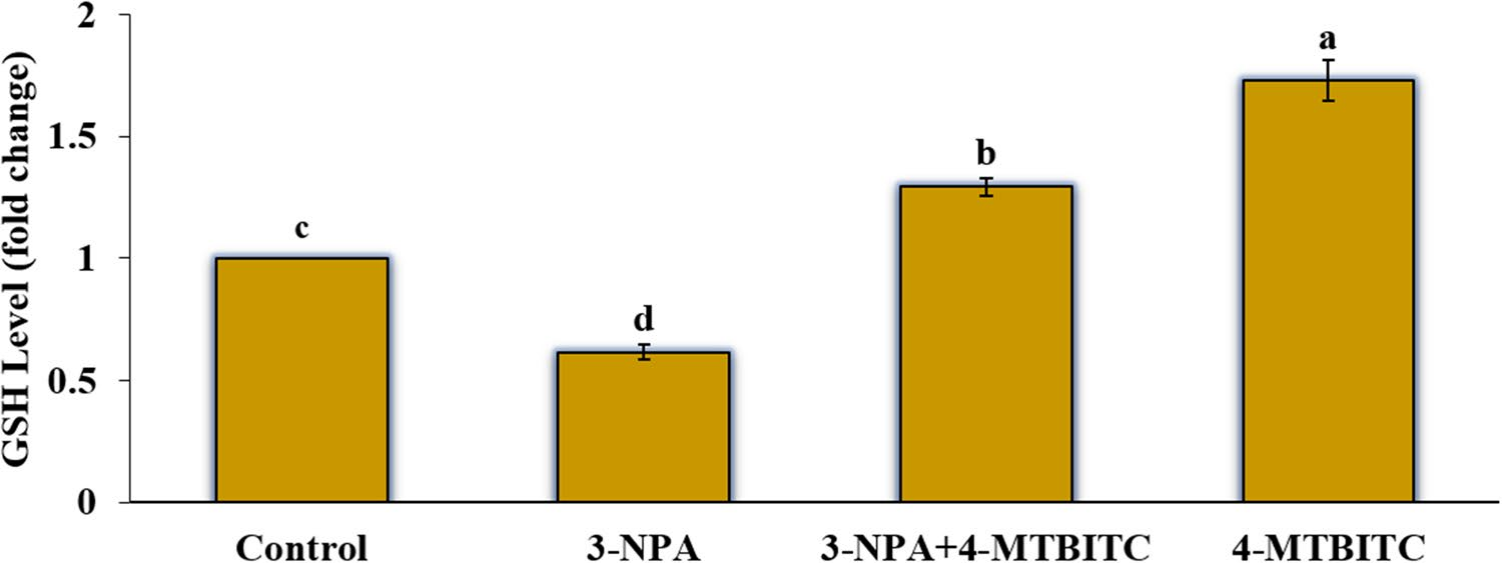
The effect of 4-MTBITC on SH-SY5Y cells treated with or without 3-NPA on the intracellular GSH level measured by using MCB fluorescent probe. Data (mean ± SEM; *p* ≤ 0.05) are expressed as fold change for three independent experiments (one-way ANOVA, followed by the Tukey post hoc test).

GSH is a significant non-enzymatic antioxidant that safeguards cells against exogenous and endogenous toxins, including ROS, and preserves the cellular redox balance. On investigation of changes in intracellular GSH level by using MCB probe, it was found to be depleted (0.61 -fold) in the cells exposed to 3-NPA neurotoxin in comparison to untreated cells. However, it significantly increased in 4-MTBITC treated cells approximately by 1.72-folds. Moreover, the pretreatment of cells with 4-MTBITC prior to 3-NPA exposure reduces the oxidative damage partly by restoring the GSH level close to control with approximately 1.29-folds increase (Fig. 4).

### Apoptotic detection in SH-SY5Y cells: morphological evidences

#### Phase-contrast microscopy

**Figure 5:**
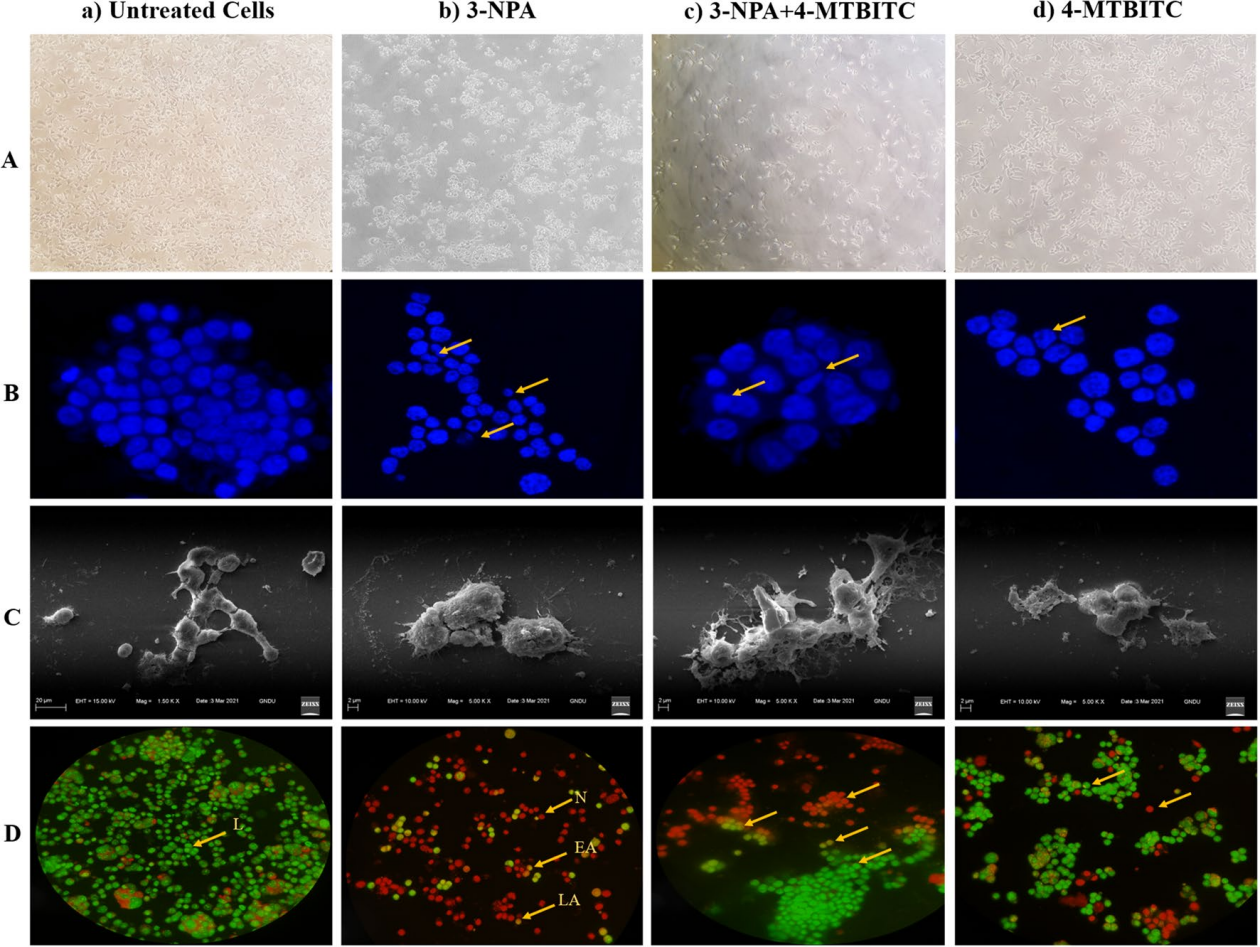
Representative microscopic images of neuroblastoma SH-SY5Y cells. (**A**) Brightfield images. (**B**) Fluorescent images of Hoechst staining showing 4-MTBITC mediated neuroprotection against SH-SY5Y cell death induced by 3-NPA exposure. (**C**) Scanning electron micrographs (SEM) of SH-SY5Y cells. (**D**) Fluorescence microscopic images of Acridine orange and ethidium bromide (AO/EtBr) double staining. Arrow indicated live (L) cells, early apoptosis (EA), late apoptosis (LA) and necrotic (N) cells in treated groups. (a) Untreated cells; (b) 3-NPA treated cells; (c) 3-NPA + 4-MTBITC treated cells; (d) 4-MTBITC alone treated cells (n = 3 coverslips per group). All images were processed using Microsoft Word “Corrections” for brightness and contrast applied equally including controls.

The changes in the cell morphology of SH-SY5Y cells, induced by 3-NPA, 4-MTBITC and their co-administration, were visualized using microscopy (Fig. 5A(a–d)). Observed morphological alterations were rounding-off of the cells, loss of neurites and detachment from the surface forming clusters in the 3-NPA treated cells (Fig. 5A(b)). 4-MTBITC treatment (Fig. 5A(d) also showed reduced viability and loss of contact of cells relative to control (Fig. 5A(a)). Whereas, Fig. 5A(c) showed the co-administration of 4-MTBITC with 3-NPA where cell survival was restored with few morphological alterations in comparison to 3-NPA treated cells.

#### Fluorescence microscopy by Hoechst staining

The characteristic fragmentation and condensation of the nuclear material in SH-SY5Y cells (arrows) appeared by Hoechst 33258 staining after treatment of 4-MTBITC (0.25 μm), with or without 3-NPA for 24 h. The images 5B(a-d) were captured with a fluorescent microscope NIKON Eclipse T*i*2. The nuclei of cells treated with 1.25 µM 4-MTBITC were found to be intact fine structure nearly similar to untreated cells. However, when SH-SY5Y cells were exposed to 3-NPA, they exhibited typical symptoms of apoptosis as nuclear DNA condensation and nuclear disintegration. The nuclear analysis of cells co-treated with 4-MTBITC and 3-NPA revealed lower apoptotic damage in contrast to the 3-NPA treated cells (Fig. 5Bc).

#### Scanning electron microscopy (SEM)

The SEM of SH-SY5Y neuronal cells revealed the marked ultrastructural changes in different groups as shown in Fig. 5C(a–d). In 3-NPA exposed cells, the evident defects were reduced size, rounding-off as well as detachment of cells from substrate, membrane blebbing and appearance of small apoptotic bodies. These defects were found to be halted to some extent in 4-MTBITC treated cells prior to 3-NPA exposure indicating the cellular protection. Collectively, the morphological alterations associated with apoptosis show that 3-NPA causes SH-SY5Y cells to undergo apoptosis, and lower dosages of 4-MTBITC ensure protection.

#### Double staining with Acridine orange and ethidium bromide (AO/EtBr)

The evidence of apoptosis is represented by double staining with Acridine orange and ethidium bromide (AO/EtBr) in 3-NPA and 4-MTBITC treated cells comparable to untreated cells. As shown in Fig. 5D(a–d), the live cells are green stained in control group whereas, nuclear changes speculated early as well as late apoptotic cell death (indicated with arrows) in 3-NPA treated SH-SY5Y cells. The lesser proportion of apoptotic nuclei is visible in 4-MTBITC treated cells (Fig. 5D(c–d)).

### Cytofluorimetric studies

#### 4-MTBITC pretreatment decreased the levels of 3-NPA induced Intracellular Reactive Oxygen Species (ROS)

**Figure 6:**
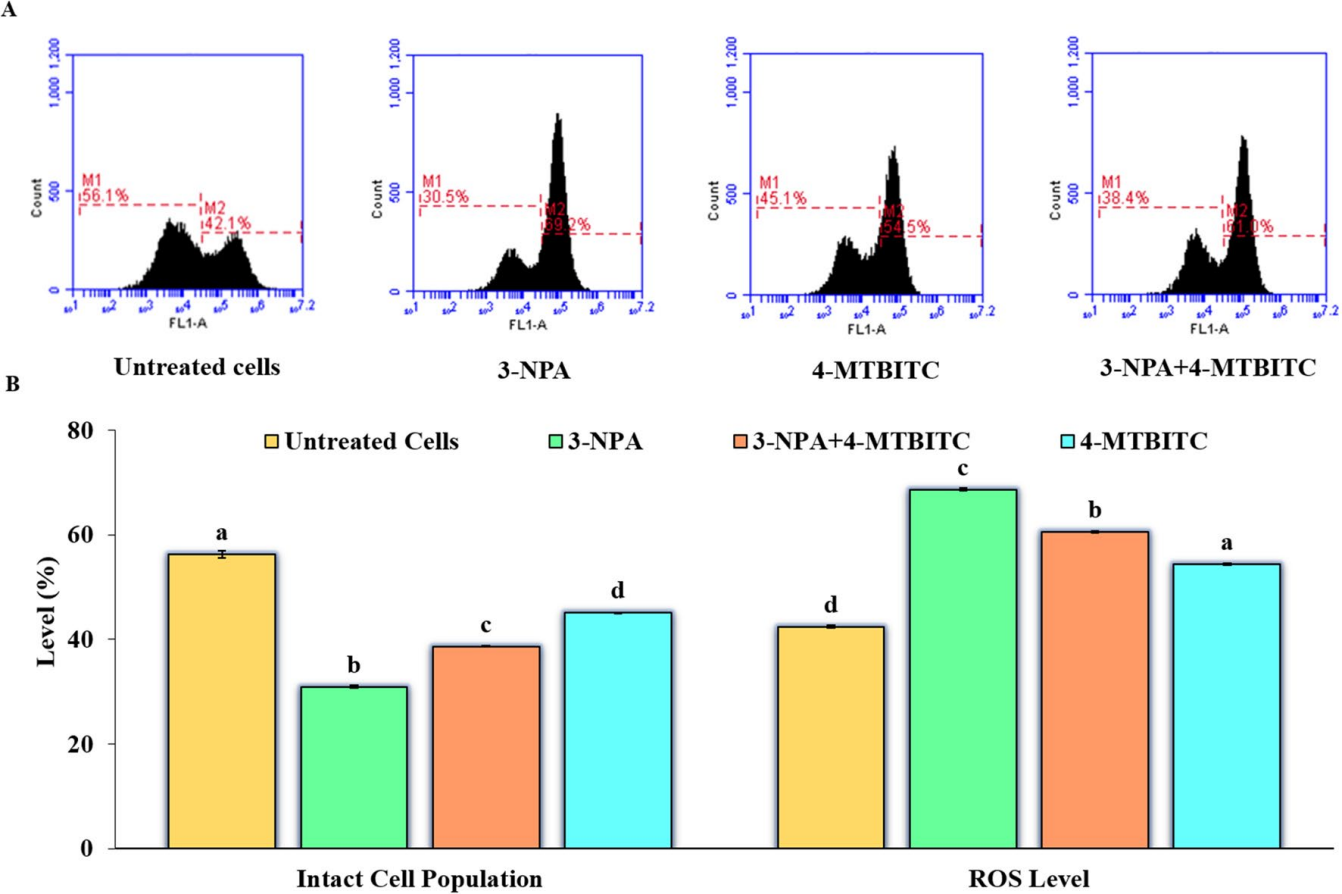
(**A**) The flow cytometric representation of Intracellular reactive species level detected by the fluorescent probe DCFH-DA. M2 represents the proportion (%) of ROS accumulated cells in comparison to live cells depicted as M1 phase. (**B**) Quantification of flow cytometry results. Results are expressed as the mean ± SE of three replicates.

The ROS production was investigated in all the experimental groups by using 2’, 7’-dichlorofluorescein diacetate (DCFH-DA) dye. The intensity of fluorescence monitored with the flow cytometer is proportional to the accumulated ROS (M2) within the cell cytoplasm (Fig. 6). The application of 3-NPA and 4-MTBITC elevated the reactive species production (M2) in SH-SY5Y cells by 69.2% and 61% respectively. However, the pre-treatment of 4-MTBITC against 3-NPA significantly defends the SH-SY5Y cells by decreasing the ROS levels by 54.5% comparable to 3-NPA exposed cells.

#### 4-MTBITC pretreatment alleviated 3-NPA-induced Mitochondrial Membrane Potential (MMP; Δψ_m_) damage in SH-SY5Y cells

**Figure 7:**
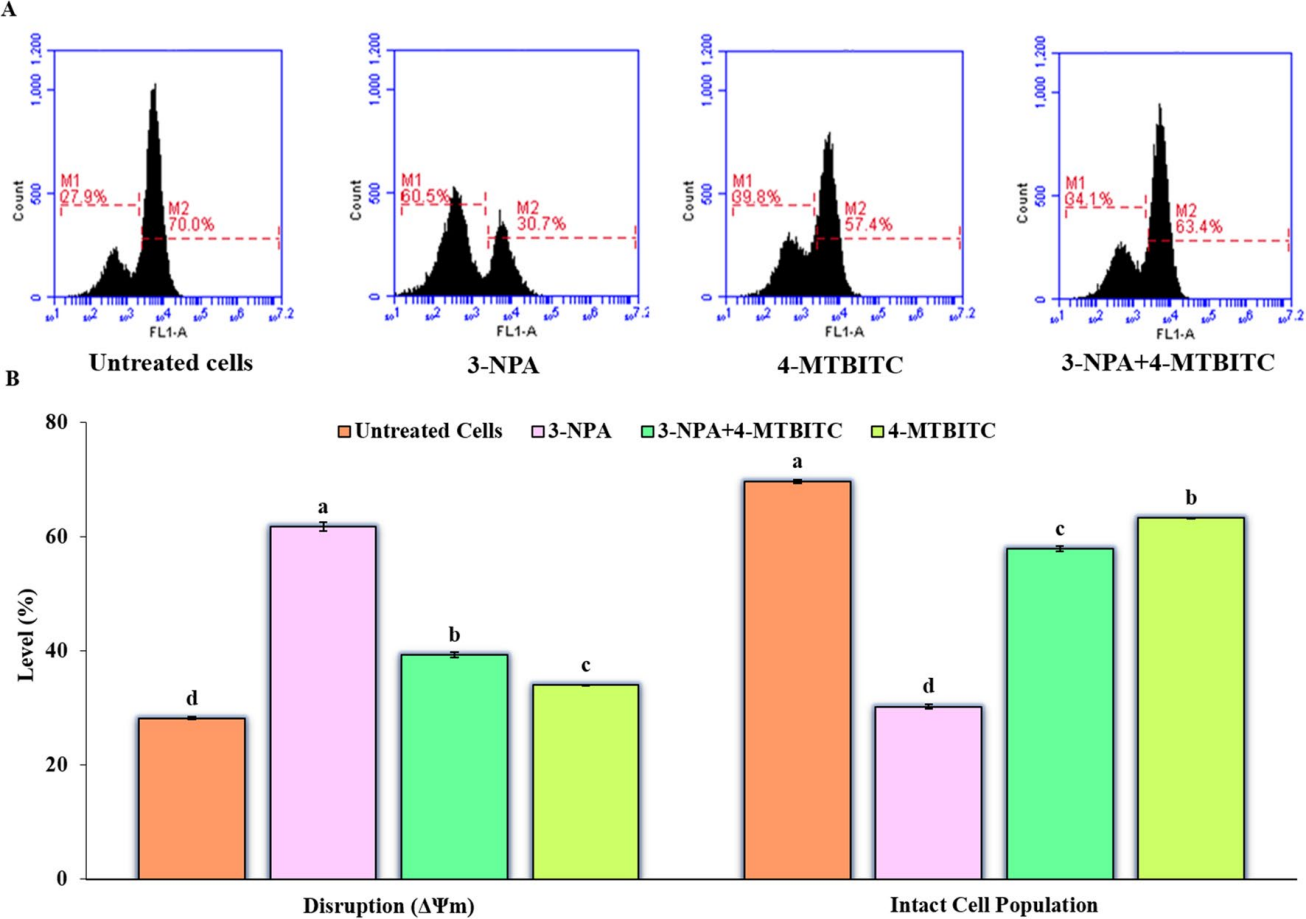
(**A**) Representative dot plots of flow cytometric loss of MMP for the 3-NPA exposed SH-SY5Y cells with or without 4-MTBITC in contrast to control cells. M1 represents the proportion (%) of cells with disrupted potential (Δψ_m_) in comparison to intact cells depicted as M2 phase (**B**) Quantification of the membrane potential and intact cell population. Values are represented as the mean ± SEM; n = 3 set of independent experiments. MMP, mitochondrial membrane potential.

The collapse of MMP was estimated by Rhodamine 123 (Rh-123) dye. In the flow cytometry dot plots, M1 represents the proportion (%) of cells with disrupted potential (Δψ_m_) in comparison to intact cells depicted as M2 phase (Fig. 7). The higher mitochondrial depolarization was evident in the 3-NPA and 4-MTBITC treated SH-SY5Y cells with approximate of 61% and 34.1% respectively. Whereas, approximately 40% mitochondrial protection was observed for 4-MTBITC (1.25 μM) against the depolarization of membrane induced by 3-NPA.

#### 4-MTBITC protects against 3-NPA-induced apoptosis in SH-SY5Y cells

**Figure 8:**
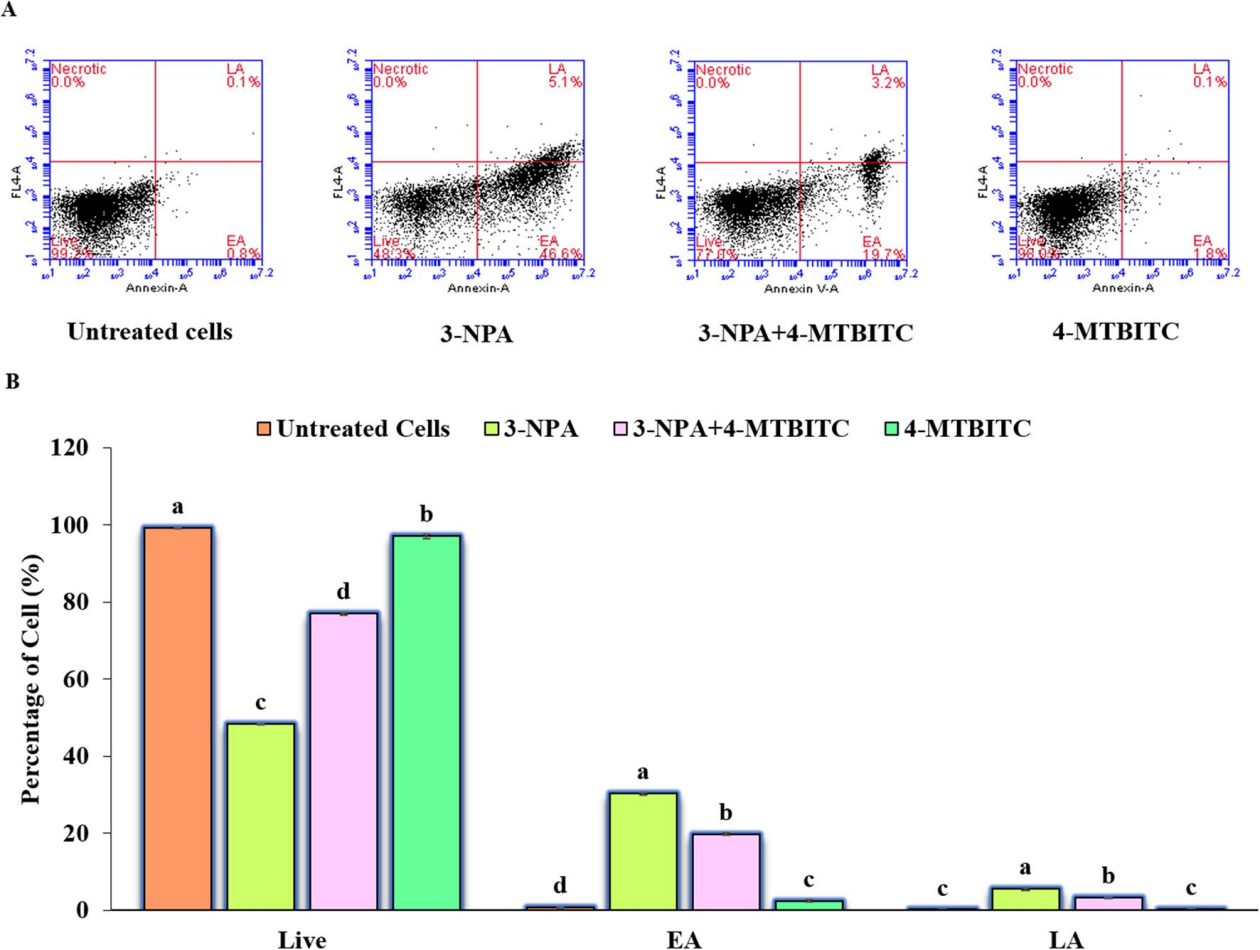
(**A**) Flow cytometric representation of apoptosis by dot plots of double annexin V/FITC-PI staining and flow cytometry in SH-SY5Y cells. Data are presented as the proportion (%) of cells in four different quadrants: live cells (Annexin V and PI negative), early apoptosis (EA = Annexin V-positive, PI negative), late apoptosis (LA = Annexin V-positive, PI positive) and necrotic (Annexin V-negative, PI positive). (**B**) The corresponding bar diagram of flow cytometry results as percentage of apoptosis. Results are expressed as the mean ± SEM of three set of independent experiments. AV, Annexin V.

The cell apoptosis analysis was performed on SH-SY5Y cells treated with 3-NPA with or without the 4-MTBITC and incubated for 24 h. The results of Annexin V-FITC/PI dual staining via flow cytometry showed the increased apoptotic rates (EA: 46.6%; LA: 5.1%) for 3-NPA induced cells compared to untreated cells (Fig. 8). The significant apoptosis of about 1.8% was also detected for such lowest concentration of 4-MTBITC (1.25 μM). However, the co-treatment of 4-MTBITC prior to 3-NPA significantly reduced the apoptosis level (EA: 19.7%; LA: 3.2%).

### Neuronal survival via BDNF/TrkB/CREB signaling, Caspase-3 down-regulation and Nrf2 induction: role of 4-MTBITC

**Figure 9:**
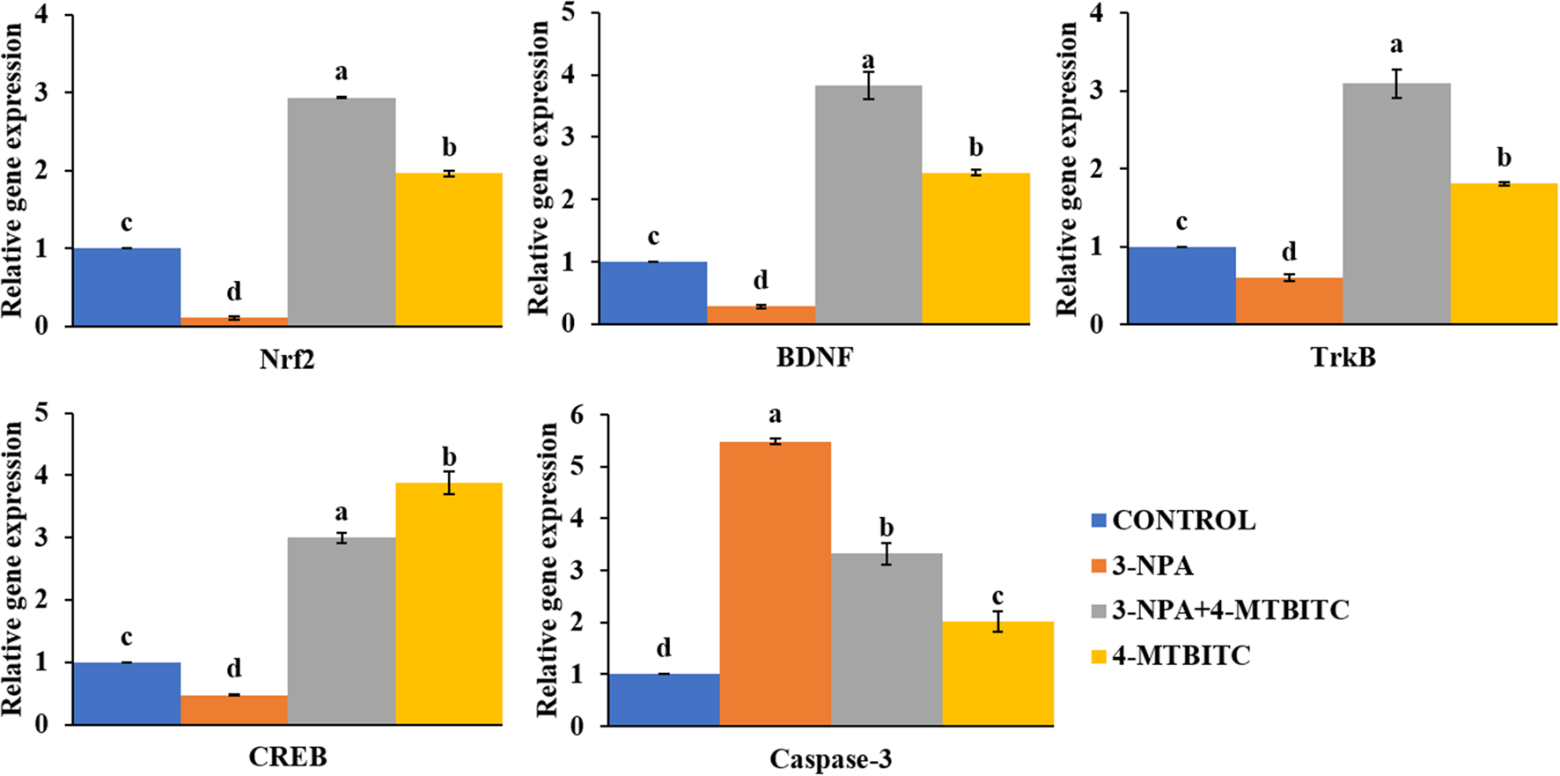
Relative gene expression of different genes Nrf2, BDNF, TrkB, CREB and Caspase-3 in SH-SY5Y cells by RT-qPCR. The values are represented as mean ± SE of three replicates and small letters as data labels indicate significant difference (*p* < 0.05) between different treatment groups.

The analysis of RT‑qPCR results revealed a significant down regulation in the expression of genes such as Nrf2, BDNF, TrkB and CREB, whereas significant upregulation of Caspase 3 at IC_50_ of 3-NPA compared to control in the SH-SY5Y cell line (Fig. 9). In contrast, the 4-MTBITC treatment counteracts the action of 3-NPA significantly by upregulating the gene expression of Nrf2, BDNF, TrkB and CREB. The obtained results with 4-MTBITC suggested the enhanced neuroprotection via BDNF as neurotrophic factor and Nrf2 as anti-oxidative factor. The significant lowered levels of Caspase-3 are consistent with supporting action of cell survival by optimal dose of 4-MTBITC. The values are presented as mean ± SE for n = 3 set of experiments.

## Discussion

Askeland and colleagues^35^ documented that HD was linked to significant nuclear DNA damage and alterations in mitochondrial features. The observed DNA damage raises the possibility that mHTT compromises genomic integrity in HD patients. Previous studies by Leba et al. have shown that in vitro DNA nicking tests enable more efficient and clinically meaningful screening of potential in vivo antioxidant agents^36^. Therefore, DNA nicking assay was used to investigate the action of 4-MTBITC as free radical scavenger against plasmid DNA subjected to both Fenton's reagent as well as 3-NPA, separately. This study is the first to use the effectiveness of 3-NPA as a nicking agent. The DNA damage was found to occur at a dose of 1 mg/ml 3-NPA for 1 h. The different doses of 4-MTBITC demonstrated its capacity to guard DNA from free radicals produced by Fenton's reagent, and that potential rises with concentration. Similarly, the results of co-administration of 4-MTBITC and 3-NPA for 1 h revealed efficacy of 4-MTBITC as a potent free radical scavenger as it established its scavenging ability in DNA nicking assay against 3-NPA induced genomic toxicity.

An increasing body of evidence highlights the tremendous potential of isothiocyanates, like SFN and 4-MTBITC, as an alternative and preventive therapy approach to limit the onset and progression of neurodegenerative illnesses^19,21,24,37^. In the current investigation, we looked for the mechanisms that would underlie the putative protective benefits of 4-MTBITC against mitochondrial malfunction and excessive oxidation stress brought by 3-NPA, a mitochondrial toxin employed in HD experimental models^38^. The neuron-like SH-SY5Y cells were exclusively chosen as the study subject since they have various essential characteristics of neurons, including the expression of dopaminergic marker genes and the physical traits of adrenergic neurons. Moreover, it is well known that post-mitochondrial processes like autophagy and apoptosis play a crucial role in inducing 3-NPA toxicity in this cell line^39^. In the present study, the cytotoxicity of 3-NPA was investigated on SH-SY5Y cells. The experimental outcomes show that the cytotoxicity of 3-NPA was dose-dependent, and IC_50_ of 3-NPA as 0.5 mM was selected for subsequent experiments (Fig. 3A). However, as shown in Fig. 3B, 4-MTBITC alone resulted in a dose-dependent drop in the metabolic ability to reduce MTT, revealing that cell survival was possible only at lower concentrations. These results support the reported findings in the literature^24,40^. Subsequent evaluation revealed that 1-h pre-treatment with 4-MTBITC considerably reduced the loss of cellular viability caused by 3-NPA over the course of 24 h (Fig. 3C). Accordingly, treatment with 4-MTBITC was able to dramatically raise (by 1.72-fold) the levels of GSH (Fig. 4). This ability of 4-MTBITC to enhance GSH levels is clear from previous experiments on human and rat non-neuronal cells as well as on neuronal cells^24,41,42^. It should be noted that when cells were pre-treated with 4-MTBITC, the considerable drop in levels of GSH seen in 3-NPA-treated cells was not observed. The fact that GSH levels in this situation (4-MTBITC + 3-NPA) were significantly lower than in cells treated simply with 4-MTBITC definitely suggests that the stimulatory actions of 4-MTBITC toward GSH levels help to clear out ROS produced following 3-NPA-mediated mitochondrial damage.

In the recent past, various studies indicated that several neurodegenerative diseases, including HD, are accompanied by mitochondrial dysfunction^43^. The research advances have demonstrated the number of beneficial pharmacological lines shielding these mitochondrial deficiencies in different neurological illnesses. However, clinical data shows that mitochondrial modulators have not been successful in showing positive effects in HD patients^44,45^. An alternate theory for this condition is that providing neuroprotection in HD may involve pharmaceutical measures to reduce adverse events referred to as post mitochondrial events caused by mitochondrial dysfunction. Decrease in MMP is a marked early apoptotic event and 3-NPA was able to reduce the MMP indicating that 3-NPA mediated cell death in SH-SY5Y cells was at least partially attributed to apoptosis^40^. In agreement with this, our in vitro data demonstrate that 4-MTBITC attenuated 3-NPA's induction of reactive species, decrease in ΔΨm, and induction of cell viability loss.

There are lots of evidences pointing to the major effect of mitochondrial complex inhibition being an increase in reactive species production^46^. Based on this and our reported results (Figs. 6 and 7), we looked into the possibility of role of antioxidant pathways in 4-MTBITC's protective actions against 3-NPA-induced cytotoxicity. Pre-treatment with 4-MTBITC totally attenuated the considerable increase in reactive species production caused by 3-NPA treatment (0.5 mM) (Fig. 6A). It is feasible to assume that the beneficial effects of 4-MTBITC in reducing the number of reactive species caused by 3-NPA may be reliant on its scavenging abilities given that such an effect was seen at lower 4-MTBITC concentrations (1.25 μM). That’s how the 3-NPA-induced negative events including the production of reactive species and a drop in MMP were hindered by 4-MTBITC pre-treatment. Moreover, the transient rise in intracellular ROS levels was observed with treatment of 4-MTBITC alone in comparison to control (Fig. 6). It is well known that induction of ROS was found to be responsible for the activation of Nrf2, which is master regulator of antioxidant defense system. Thereby, increasing the intracellular GSH as a part of protective entity against 3-NPA toxicity^47^. Additionally, the literature supports that GSH further interacts with ROS and acts as a substrate for the activation of downstream antioxidant defense against oxidative damage^48^. Thus, the initial rise of ROS as well as enhanced GSH content at the experimental dose of 4-MTBITC may be attributed to the adaptive neuroprotective mechanism of isothiocyanates for maintaining the redox cellular homeostasis^21,24^. The further investigation of mechanistic action of 4-MTBITC was done by RT-qPCR. Keeping in mind the possible potency of 4-MTBITC for different endogenous targets, the analysis of gene expression levels of Nrf2, BDNF, TrkB, CREB and Caspase-3 was carried out in the present study. The results demonstrated that 4-MTBITC reduced the oxidative stress caused by 3-NPA in SH-SY5Y cells by upregulating the expression of transcription factor Nrf2. The previous studies have reported that the expression of Nrf2 tends to decline as the disease progresses, it is upregulated in the primary stages of HD by the creation of reactive species brought on by the toxicity of 3-NPA. The protective impact of Nrf2 against oxidative insults may be greatly enhanced by the stimulation of transcription of protective genes involved in antioxidative potential, such as HO-1 synthetase^49^. Additionally, Zhang et al. mentioned the requirement of Nrf2 in stress-induced neurogenesis and how up-regulation can lessen the impact of mitochondrial malfunction, control the generation of ROS, and lessen neuronal apoptosis^50^.

The motor, cognitive, and psychiatric decline are primarily caused by the dysfunction and mortality of the medium sized spiny neurons (MSNs) of the striatum and this selective susceptibility of MSNs in HD has been attributed to a deficit in BDNF/TrkB signalling^51^. According to Tejeda et al., HD patients have been found to have lower levels of striatal BDNF protein, which is caused by decreased neurotrophin expression and impaired corticostriatal transportation^52^. BDNF has been shown to play a major role in learning and memory and is regarded as a key synaptic regulator of synapse development and synaptic plasticity. BDNF activates intracellular signaling pathways by binding to the particular receptor TrkB, which results in receptor dimerization and autophosphorylation^53,54^. In consistent to these investigations, the 4-MTBITC treatment against 3-NPA induced toxicity significantly boosted BDNF expression and thus may potentially confer neuroprotection. Furthermore, growing data suggests that BDNF-mediated neuronal activity may be the root cause of AKT activation. AKT signaling pathway can be activated by persistent BDNF induction^55^. The AKT pathway, one of the most well studied cellular signaling pathways, has a strong connection to HD's pathogenesis and is very vulnerable to oxidative stress^56^. Target genes are activated by an active transcription complex created by the action of AKT, which can also cause the phosphorylation of CREB. A transcription factor called CREB is crucial for neurogenesis and neural plasticity^57^. Aside from the fact that oxidative stress exposure is directly linked to a decrease in CREB expression, research have suggested that CREB phosphorylation is decreased during the clinical pathophysiology of HD and that CREB phosphorylation is increased during the anti-HD response in people^52,58^. BDNF is also vital CREB target, and CREB activation is necessary for BDNF expression. These results imply that the HD pathogenesis may be closely related to the BDNF/TrkB/CREB cycle signalling system^59,60^. Despite the fact that current findings suggested that 4-MTBITC had no effects on the protein expression in the BDNF/TrkB/CREB signalling pathway in normal SHSY5Y cells, the situation was very different under the condition of oxidative stress, where 3-NPA-triggered decreased phosphorylation of TrkB, AKT, and CREB as well as reduced expression of BDNF could be reversed by 4-MTBITC. In constrast, the activation of downstream targets, such as the anti-apoptotic protein Caspase-3, may come from the overexpression of CREB and prevent neuronal apoptosis^61,62^. Similar results were obtained in the current study, where 4-MTBITC reduced the 3-NPA-induced Caspase-3 activation and delayed apoptotic neuronal death. Moreover, the effects of 4-MTBITC on ROS generation and MMP deficit were dependent on the stimulation of the BDNF/TrkB/CREB signaling, suggesting that this pathway is likely to be a crucial stage in the development of 4-MTBITC's anti-oxidative effects.

## Conclusion

**Figure 10:**
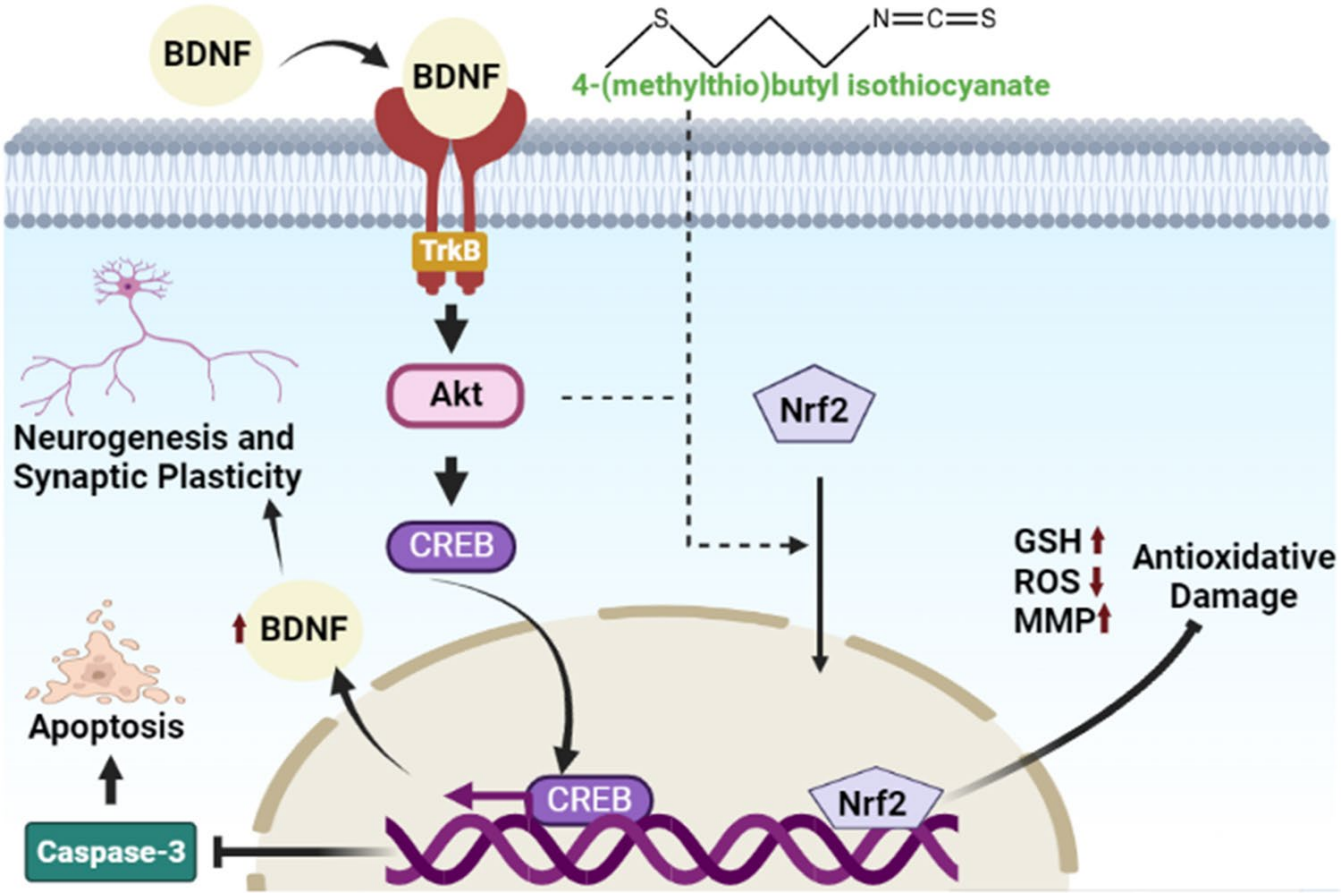
Diagrammatic representation summarizing the key findings of the study. 4-MTBITC ensuing neuromodulatory action against the 3-NPA-induced cytotoxicity via activation of BDNF/TrkB/CREB signalling and the downstream targets, such as the anti-apoptotic protein Caspase-3. Also, the 4-MTBITC induced Nrf2 upregulation lessen the impact of mitochondrial malfunction and control the generation of ROS, thereby, conferring cytoprotection..

According to this study, the 3-NPA is a pBR322 DNA nicking agent and 4-MTBITC is a powerful free radical scavenger against 3-NPA-induced DNA damage. Moreover, the present study provided the first evidence that dietary phytoconstituent 4-MTBITC has the neuroprotective effects on 3-NPA induced cytotoxicity in cultured human dopaminergic SH-SY5Y cells. The outcomes showed that 4-MTBITC treatment reduced the redox impairment, apoptotic state and mitochondrial dysfunction caused by 3-NPA in SH-SY5Y cells. Additionally, it may function to strengthen the antioxidant system, stimulates BDNF/TrkB/CREB signaling, and supports neuronal survival (Fig. 10).

## Material and methods

### Chemicals and reagents

Dulbecco's Modified Eagle Media (DMEM) and Ham’s F12 Media (DMEM/Ham’s F12) and 3-(4,5-dimethylthiazol-2-yl)-2,5-diphenyltetrazolium bromide, MTT were purchased from Himedia Lab, Mumbai, India. 3-NPA, DNase I, L-Glutamine, Fetal bovine serum (FBS), ethidium bromide, penicillin, streptomycin, acridine orange, 2,7-dichloro dihydro fluorescein diacetate (DCFH-DA), glutaraldehyde, propidium iodide, rhodamine 123 (Rh123), DAPI, annexin V-FITC apoptosis detection kit and fluoromount were bought from Sigma-Aldrich (St. Louis, MO, USA). The iScript cDNA synthesis kit was purchased from Bio-rad, California, USA.

### Isolation and characterization of 4-(methylthio)butyl isothiocyanate (4-MTBITC)

The seeds of plant *Eruca sativa* variety named RTM2002 were purchased from Sri Karan Narendra Agriculture University, Jobner, Rajasthan, India. The plant material was further identified and authenticated by Herbarium, Department of Botanical and Environmental Sciences (DOBES), Guru Nanak Dev University, Amritsar with voucher number 7297. The use of plant material in the current study complies with the international, national, and institutional guidelines. Following a standard protocol established in our lab by Arora et al. (2018), the hydro distillation method using the Clevenger apparatus was used to extract the 4-MTBITC from the seeds of *Eruca sativa*^27^. To one liter of double distilled water in the flat bottom flask, 100 g of crushed seeds of *E. sativa* along with a magnetic bead were added. The flask was placed on a hot plate with a magnetic stirrer and boiled for 3 h. The mixture of oil and water was put in the separating funnel and oil was extracted with solvent dichloromethane (DCM). The DCM was recovered in a rotary evaporator at 30ºC to obtain the crude oil which was kept at -40ºC storage. The collected phytochemical was characterized using UHPLC (Shimadzu, Japan), Mass spectrometry (LC-5050, Shimadzu) and Nuclear Magnetic Resonance (NMR) spectroscopy (500 MHz, Brucker).

### DNA nicking assay

DNA free radical scavenging assay was performed to evaluate ability of 4-MTBITC to protect supercoiled pBR322 DNA from the devastating effects of the Fenton reagent that produces the hydroxyl radicals. 0.5 μl of plasmid DNA was mixed with 10 μl of Fenton’s reagent (30 mM H_2_O_2_, 50 μM ascorbic acid, and 80 μM FeCl_3_) followed by the addition of 10 μl of 4-MTBITC (0.3, 0.6 and 0.9 mg/ml)^28^. Using distilled water, the final volume of the mixture was brought to 20 μl. The reaction mixture was incubated at 37 °C for 30 min and the sample was loaded on 1% agarose gel (prepared by dissolving 0.5 g agarose in 50 ml 1X TBE buffer) and electrophoresed followed by staining with ethidium bromide. Rutin was taken as a positive control. The gel was observed under the Gel Doc XR system and quantification was done by using LabImage 1D software (Kapelan Bio-Imaging, Leipzig, Germany).

### Cell culture and treatment

SH-SY5Y cell line was purchased from the National Centre for Cell Science (NCCS), Pune, India. The cells were routinely grown in T-25 flask containing a mixture of Dulbecco’s Modified Eagle Media (DMEM) and Ham’s F12 Media (DMEM/Ham’s F12) in ratio 1:1, enriched with 10% fetal bovine serum, streptomycin (100 μg/ml), penicillin (100 U/ml) and 1% 2 mM nonessential amino acid (L-Glutamine). The cells were kept at 37 °C in a humified CO_2_ incubator having 5% CO_2_. Cells were further sub-cultured at 70–80% confluences and media was regularly replaced with fresh media.

3-NPA was uniformly solubilized in phosphate buffer saline (PBS, pH 7.4). 4-MTBITC was dissolved in 0.05% DMSO. To study the neuroprotective effect against 3-NPA induced neurotoxicity, cells were pretreated with 4-MTBITC for 1 h.

### 3-(4,5-Dimethylthiazol-2-yl)-2,5-diphenyl tetrazolium bromide (MTT) assay

#### Determination of neurotoxicity of 3-NPA

The neuroblastoma (SH-SY5Y) cells were cultured in 96-well plates to assess the cell viability in terms of 3-(4,5-dimethyl-2-thiazolyl)-2,5-diphenyl-2*H*-tetrazolium bromide (MTT) reduction to formazan. To determine the cytotoxicity induced by 3-NPA, cells were treated with different doses of 3-NPA (0.1–15 mM) for 24 h. The more the amount of formazan, higher is the viability of cells. The MTT (5 mg/ml) dissolved in PBS was added to media for 4 h. After incubation at 37 °C in 5% CO_2_, the media was discarded and crystals of formazan were solubilized in DMSO (100 μl). The crystals of formazan were quantified at 570 nm using a microplate reader. The toxicity of 3-NPA was determined as a percent of decrease in intracellular granules of MTT.

#### Determination of neuroprotective activity of 4-MTBITC

To estimate the cytoprotective potential of 4-MTBITC, SH-SY5Y cells were subjected to different doses of 4-MTBITC for 24 h. Further, to determine the optimal dose at which 4-MTBITC protects cell death, the 3-NPA (IC_50_) induced cells were pretreated with different concentrations of 4-MTBITC (0.625–1000 μM) for 1 h. The neuroprotective activity in terms of increase in intracellular MTT granules was measured by MTT formazan exocytosis assay as explained above. The absorbance of DMSO soluble MTT was measured at 570 nm. Data were expressed as a percentage of untreated control cultures.

### Experimental design

After estimating neurotoxicity induction by 3-NPA and optimal protective concentration of 4-MTBITC, the following experimental design was set up.

- *Group I*: Control treated with DMSO (0.05%).
- *Group II*: 3-NPA (0.5 mM).
- *Group III*: 3-NPA (0.5 mM) + 4-MTBITC (1.25 µM).
- *Group IV*: 4-MTBITC alone (1.25 µM).

### Estimation of GSH level

The glutathione (GSH) levels were determined in SHSY-5Y cells (2 × 10^5^ cells/ml) seeded in 6-well plates and incubated in CO_2_ incubator (humidified 5%) for 24 h at 37 °C by modified method of^29^. The SH-SY5Y cells were subjected to different experimental doses. After the incubation, the treatment of each well was replaced with 100 μl of monochlorobimane (MCB), a fluorescent probe of concentration 50 μM. Subsequently, after keeping samples for 30 min at ambient temperature, the levels of GSH were estimated using a multiplate reader with excitation at 355 nm and emission at 460 nm. The readings were determined as concentrations of GSH (μM) in terms of fold increases comparable to untreated cells.

### Morphological studies for apoptotic detection

The SH-SY5Y cells after treatment with 3-NPA with or without 4-MTBITC were observed for various morphological modifications under the phase-contrast microscope, scanning electron microscope (SEM) and fluorescence microscope.

#### Phase-contrast microscopy

The SH-SY5Y cells exposed to different experimental treatments were monitored and morphological changes were detected under a phase-contrast microscope, Nikon Eclipse T*i*2, Japan.

#### Scanning electron microscopy (SEM)

SH-SY5Y cells (4 × 10^5^ cells/well) were plated overnight over 12 mm circular coverslips and subjected to different experimental doses based on the method given by Chen et al.^30^. After that cells were harvested and subsequently fixed with a mixture of 4% paraformaldehyde (PFA) and 2.5% glutaraldehyde for 4 h at − 20 °C. Dehydration of cells was done with a series of chilled ethanol for time period of 5 min. The cell-adhered coverslips were mounted on the stubs and coated with silver using sputter coater (Quorum Q150R ES). The images were captured with a SEM, Carl Zeiss, EVO LS10, Germany.

#### Fluorescence microscopy

The morphology of nucleus in SH-SY5Y cells was analyzed for various alterations by Hoechst staining^31^. The SH-SY5Y cells (4 × 10^5^/well) were cultured in six-well plate with coverslips till confluency (70–80%). Thereafter cells were treated with different experiment doses. After that, cells were twice washed with cold PBS and incubated with 4% paraformaldehyde for about 30 min in dark. The cells were washed with PBS followed by staining with DAPI (10 μg/ml) dye and kept for time period of 15 min under dark conditions. After that, cells were twice washed to remove traces of dye with cold 1 × PBS and slides were mounted using fluoromount. The morphological changes of nucleus were imaged under a fluorescent microscope (Nikon Eclipse T*i*2, Japan).

#### Acridine orange and ethidium bromide (AO/EtBr) double staining

To estimate the process of cell death, fluorescence microscopy with AO/EtBr dual staining was done as per the protocol recommended by Liu et al.^32^. SH-SY5Y cells (4 × 10^5^/well) were grown in T-25 flask and after confluency, cells were treated with different doses. After 24 h, suspended and adherent cells were trypsinized and harvested to make pellet cells were centrifuged at 2500 rpm for 5 min. The cell pellet was then mixed with PBS (100 μl). From these cell suspensions, 25 μl of cells suspension mixed with 5 μl of dual fluorescent staining solution (100 μg/ml AO and 100 μg/ml EB (AO/EB)) and placed on glass slides and then covered with a coverslip. The morphology of apoptotic and necrotic cells was immediately assessed using a fluorescence microscope.

### Cytofluorimetric studies

#### Intracellular reactive oxygen species (ROS)

The ROS formation was assessed using fluorescent probe 2′,7′-dichlorodihydroflurescein diacetate, named DCFH-DA according to method recommended by LeBel et al.^33^. The SHSY-5Y cells (4 × 10^5^ cells/well) were incubated with different treatment groups. After 24 h, the cells were incubated with the DCFH-DA (10 μg/mL) dye for 30 min. The cells were harvested after trypsinization and centrifuge at 2500 rpm to obtain pellet. The pellet was suspended into PBS and intracellular levels of ROS were estimated by using C6 Flow Cytometer (BD Accuri TM) and the data was determined as % intracellular reactive species. The experiment was performed in triplicates and BD Biosciences software was used to analyze the results.

#### Mitochondrial membrane potential (MMP; Δψ_m_)

To check the MMP variations in SHSY-5Y cells, the cells were incubated with different doses in 24-well plates and were analyzed as per the method suggested by Carlson and Ehrich^34^. After incubation, the cell pellets of all the groups were obtained using trypsinization and centrifugation at 2500 rpm for 4 min. This was followed by cell fixation with 70% of chilled ethanol and cell staining using 2 µg/ml of Rhodamine 123 (Rh-123) for time period of 30 min. The SH-SY5Y cells were washed thrice using PBS and analyzed using the flow cytometer.

#### Annexin V-Fluorescein isothiocyanate (FITC) staining

The SH-SY5Y cells were cultured till confluency and treated with various experimental doses. The trypsinized cells were washed, centrifuged and dissolved in the binding buffer (Annexin V-FITC Apoptosis Detection Kit (Sigma)). Further, in the cell suspension (500 µL), 5 µL of annexin-V-FITC and 10 µL of propidium iodide (PI) dyes were mixed and gently vortexed followed by incubation in dark for 10 min at ambient temperature. Finally, the analysis was carried out on AccuriTMC6 Flow Cytometer.

### Analysis of gene expression using Quantitative Real‑Time PCR (RT‑qPCR)

RNA was isolated from SH-SY5Y cells exposed to 3-NPA with or without 4-MTBITC using Trizol Reagent. The extracted RNA was mixed in TE buffer and kept at 60 °C for 5 min to avoid any DNA impurity, samples were incubated with DNase I for 30 min at room temperature. The OD of isolated RNA was observed by Nano-Drop spectrophotometer at 260 nm and 280 nm. Then, same quantity of RNA was utilized for the preparation of complementary DNA using iScriptTM synthesis kit. The quantification of cDNA was performed and similar amount was further used for performing RT-qPCR by iQSYBR Supermix system. The sequence of particular primers (Nrf2, BDNF, TrkB, CREB and Caspase-3) taken for analysis are given in Table 1. To estimate the relative expression, the RT-qPCR reaction was carried out and data was quantified using the ΔΔC_T_, comparative threshold cycle method. β-actin was taken as housekeeping gene. The C_T_ value of each target gene was normalized by C_T_ value of housekeeping gene, β-actin. The relative expression of genes was represented as 2^−ΔΔCT^ ± SE.

**Table 1:** The list of RT-qPCR primers and their sequences.

S. No	Primer	Accession No	Product length	Sequence of Oligonucleotides (5′-3′)	Source
1	Nrf2	NM_006164.5	118	Forward: AGGTTGCCCACATTCCCAAA Reverse: AGTGACTGAAACGTAGCCGA	NCBI
2	BDNF	NM_001143812.2	142	Forward: GAAAGCTAGGGGAGCGAGAC Reverse: CTTCGAGGGGTGTTCCAGC	NCBI
3	TrkB	S76473.1	124	Forward: AAAGAAGAAGCCGCAAAGCG Reverse: GGGTCCATGCCACCTTATCC	NCBI
4	CREB	NM_001382431.1	186	Forward: CAGTGGGACAGAGGAGCAAG Reverse: AAGGTCAAGTGCTACCGTGG	NCBI
5	Caspase 3	NM_001354777.2	176	Forward: CTCCTAGCGGATGGGTGCTATTG Reverse: TTATTAACGAAAACCAGAGCGCCG	NCBI
6	β-actin	NM_001101.5	72	Forward: AGACCTGTACGCCAACACAG Reverse: TTCTGCATCCTGTCGGCAAT	NCBI

### Statistical analysis

Data was expressed as Mean ± standard error (SE) and statistically analyzed for variances and interactions using one-way analysis of variance (ANOVA). Statistical evaluation was performed in triplicates and *p* ≤ 0.05 were considered significant.

## Supplementary Information

Supplementary Information.

## Data Availability

The datasets used and/or analysed during the current study are available from the corresponding author on reasonable request.

## Abbreviations

HD: Huntington’s disease
3-NPA: 3-Nitropropionic acid
4-MTBITC: 4-(Methylthio)butyl isothiocyanate
BDNF: Brain-derived neurotrophic factor
TrkB: Tyrosine kinase B
CREB: CAMP response element-binding protein
Nrf2: Nuclear factor erythroid 2–related factor 2
WHO: World Health Organization
NDDs: Neurodegenerative diseases
ROS: Reactive oxygen species
Htt: Huntingtin protein
GSH: Glutathione
SFN: Sulforaphane
DMEM: Dulbecco’s Modified Eagle Media
DMEM/Ham’s F12: Dulbecco’s Modified Eagle Media (DMEM) and Ham’s F12 Media
MTT: 3-(4,5-Dimethylthiazol-2-yl)-2,5-diphenyltetrazolium bromide
FBS: Fetal bovine serum
DCFH-DA: 2,7-Dichloro dihydro fluorescein diacetate
Rh123: Rhodamine 123
UHPLC: Ultra-High Pressure Liquid Chromatography
NMR: Nuclear Magnetic Resonance
NCCS: National Centre for Cell Science
PBS: Phosphate buffer saline
FR: Fenton’s reagent
MCB: Monochlorobimane
SEM: Scanning electron microscopy
PFA: Paraformaldehyde
AO/EtBr: Acridine orange and ethidium bromide
MMP: Mitochondrial membrane potential
FITC: Annexin V-Fluorescein isothiocyanate
PI: Propidium iodide
RT‑qPCR: Real-time quantitative polymerase chain reaction
SE: Standard error
ANOVA: One-way analysis of variance
