# Supplementary material for: Neuromodulatory effect of 4-(methylthio)butyl isothiocyanate against 3-nitropropionic acid induced oxidative impairments in human dopaminergic SH-SY5Y cells via BDNF/CREB/TrkB pathway
Source: Sci Rep. 2023 Mar 17;13:4461. doi: 10.1038/s41598-023-31716-3 (PMC10023800; doi:10.1038/s41598-023-31716-3)
Supplement: Supplementary file 1 — Supplementary Information. [file 41598_2023_31716_MOESM1_ESM.pdf]

Supplementary Information File

Neuromodulatory effect of 4-(methylthio)butyl Isothiocyanate against 3-Nitropropionic Acid Induced Oxidative Impairments in Human Dopaminergic SH-SY5Y Cells via BDNF/CREB/TrkB pathway

Prabhjot Kaur[1], Shivani Attri[1], Davinder Singh[1,2*], Farhana Rashid[1], Sharabjit Singh[1], Avinash Kumar[1], Harjot Kaur[3], Neena Bedi[4], Saroj Arora[1*]

[1]Department of Botanical and Environmental Sciences, Guru Nanak Dev University, Amritsar-143005, India

[2]Department of Molecular Genetics, The Ohio State University, Columbus, OH-43210, USA

[3]Department of Biotechnology, Punjabi University, Patiala-147001, India

[4]Department of Pharmaceutical Sciences, Guru Nanak Dev University, Amritsar-143005, India

[*]Correspondence to:

Dr. Saroj Arora, Department of Botanical and Environmental Sciences, Guru Nanak Dev University, Amritsar-143005, India

Email: sarojarora.gndu@gmail.com; dr.sarojarora@gmail.com

[*]Co-Correspondence to:

Dr. Davinder Singh, Department of Molecular Genetics, The Ohio State University, Columbus, OH-43210, USA; Department of Botanical and Environmental Sciences, Guru Nanak Dev University, Amritsar-143005, India

Email: sidhu.davinder0001@gmail.com; singh.1900@osu.edu

This file contains following sections:

(1) Information about plant used
(2) Supplementary figures: Figure S1
(3) Raw full-length gel images of gels used in this manuscript: Raw images of gel 1 and 2
(4) Supplementary methods sections: Section 1

1. Information about the plant used in the present study:

Plant used: *Eruca sativa*
Scientific Classification:
Kingdom: Plantae
Order: Capparales
Family: Brassicaceae
Genus*: Eruca*
Species*: sativa*

2. Supplementary figures: Figure S1

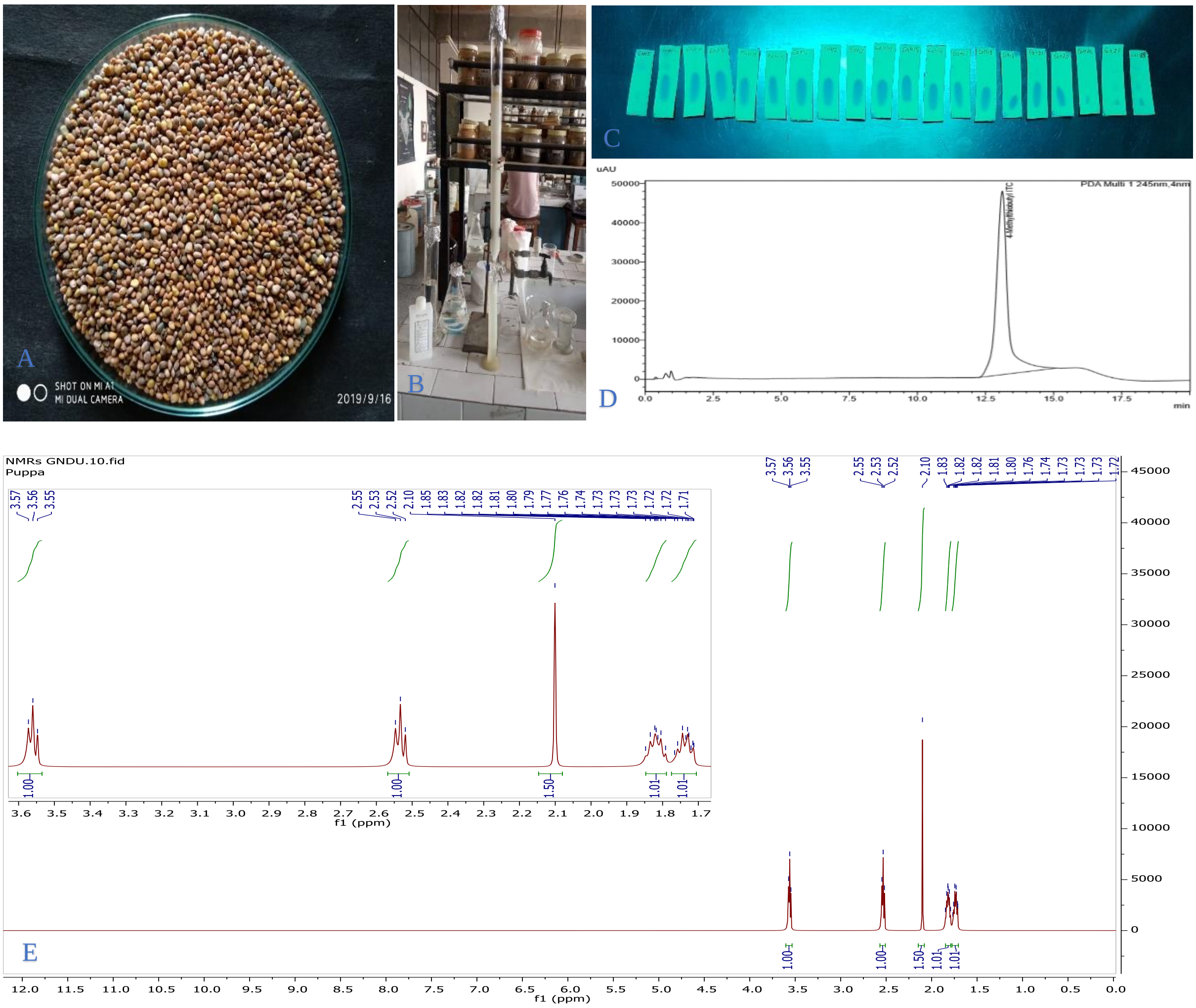

Figure S1: Characterization of 4-methylthiobutyl isothiocyanate (4-MTBITC) isolated from *Eruca sativa* (A): Seeds of *Eruca sativa*, (B): Column chromatography setup, (C): Thin layer chromatogram of different fractions, (D): ^{1}H NMR spectrum of isolated 4-MTBITC ($CDCl_3$) δ 3.56, 5.55-5.52, 2.10, 1.85-1.79, 1.77-1.71

3. Raw full-length gel images of electrophoresis gels used for DNA nicking assay

Figure 1

Raw files supporting Figure 1(A)

1 2 - 4 5 - 7 8

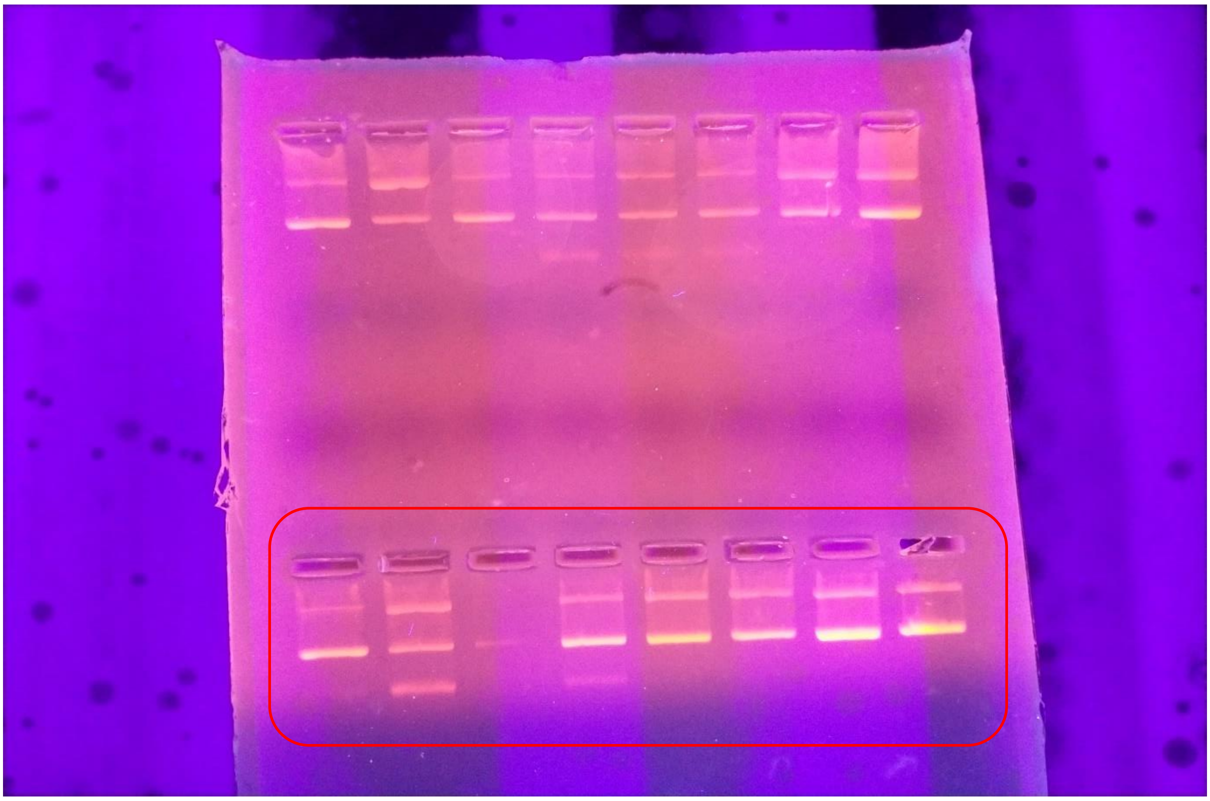

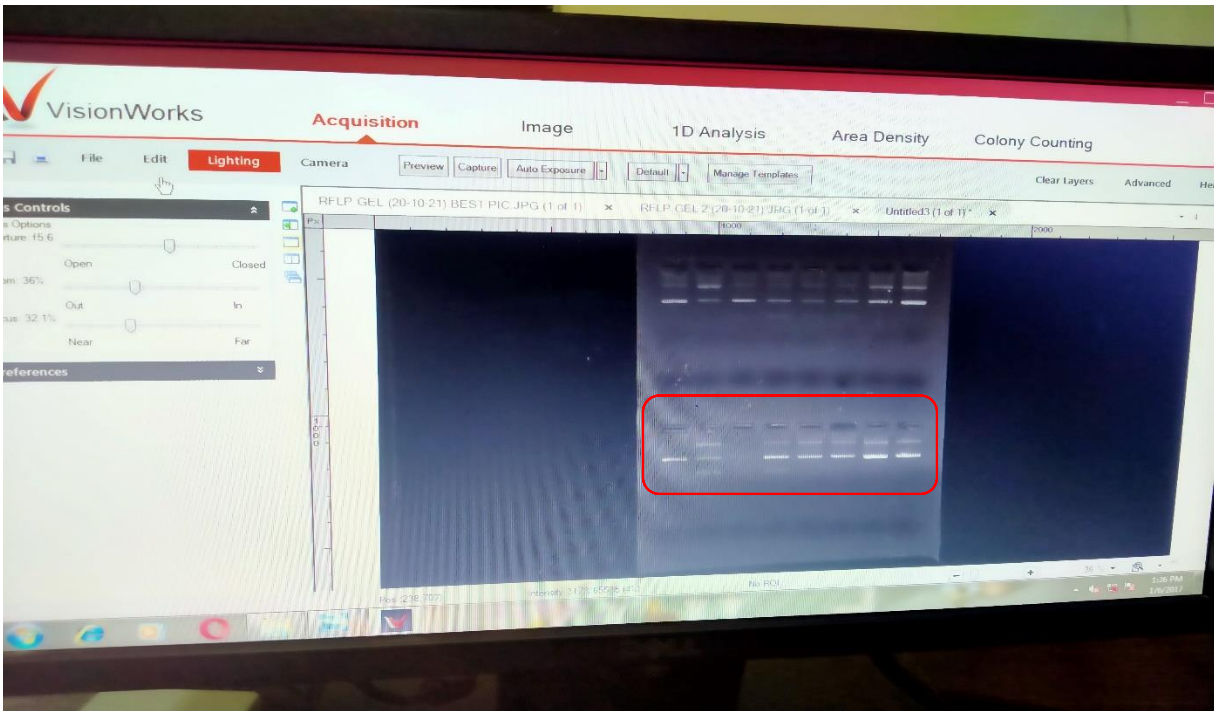

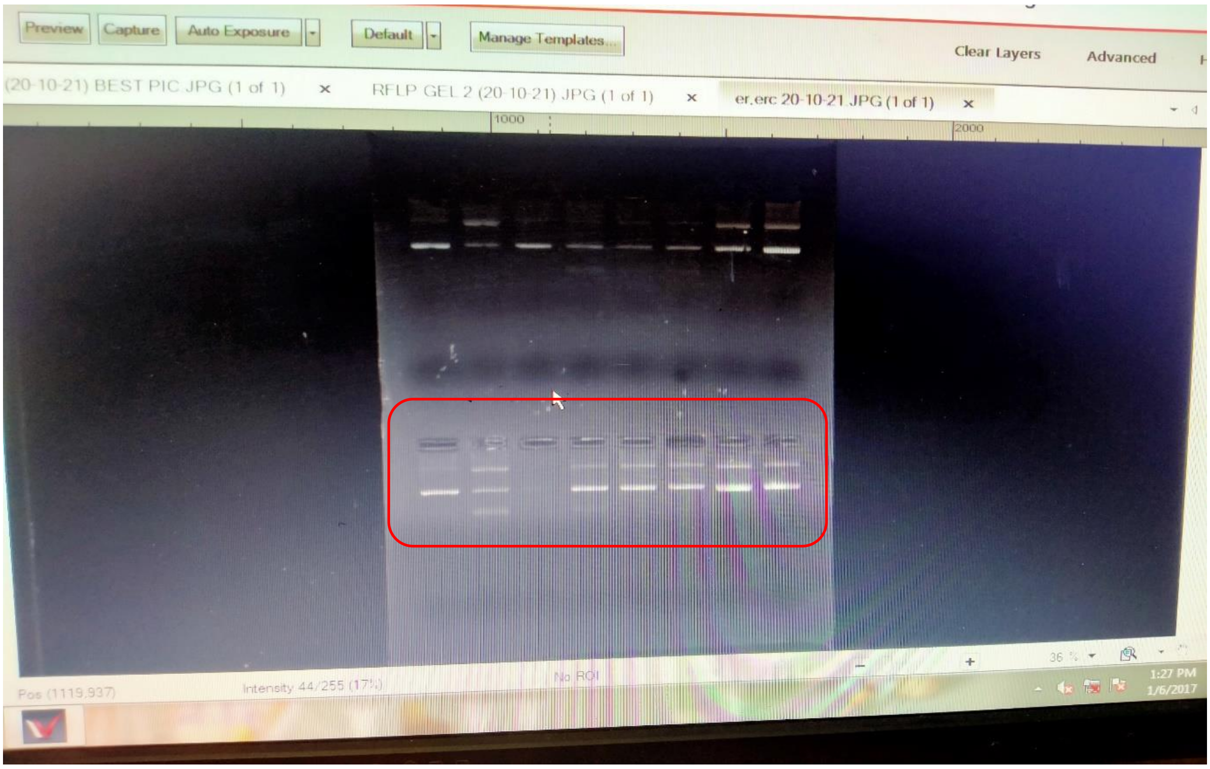

Cropped Figure 1(A)

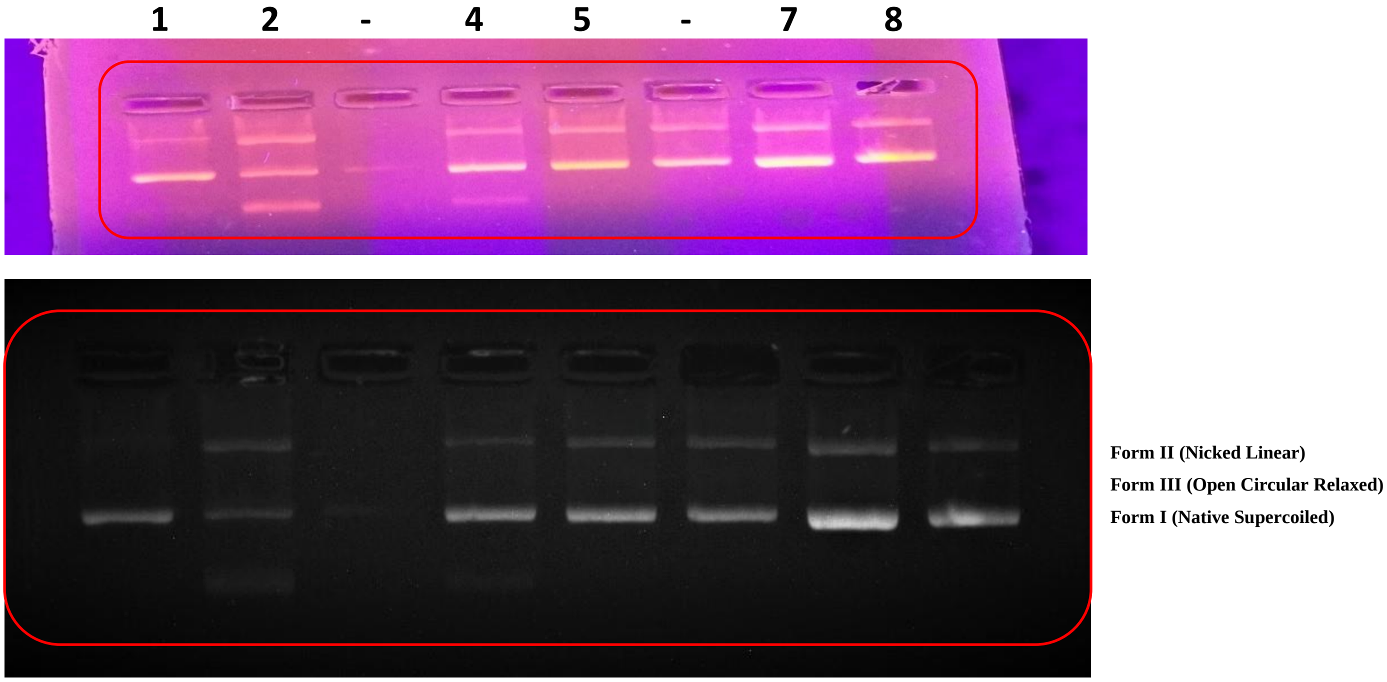

Figure 2

Raw files supporting Figure 2(A)

1 2 3 4 5 6 7 8

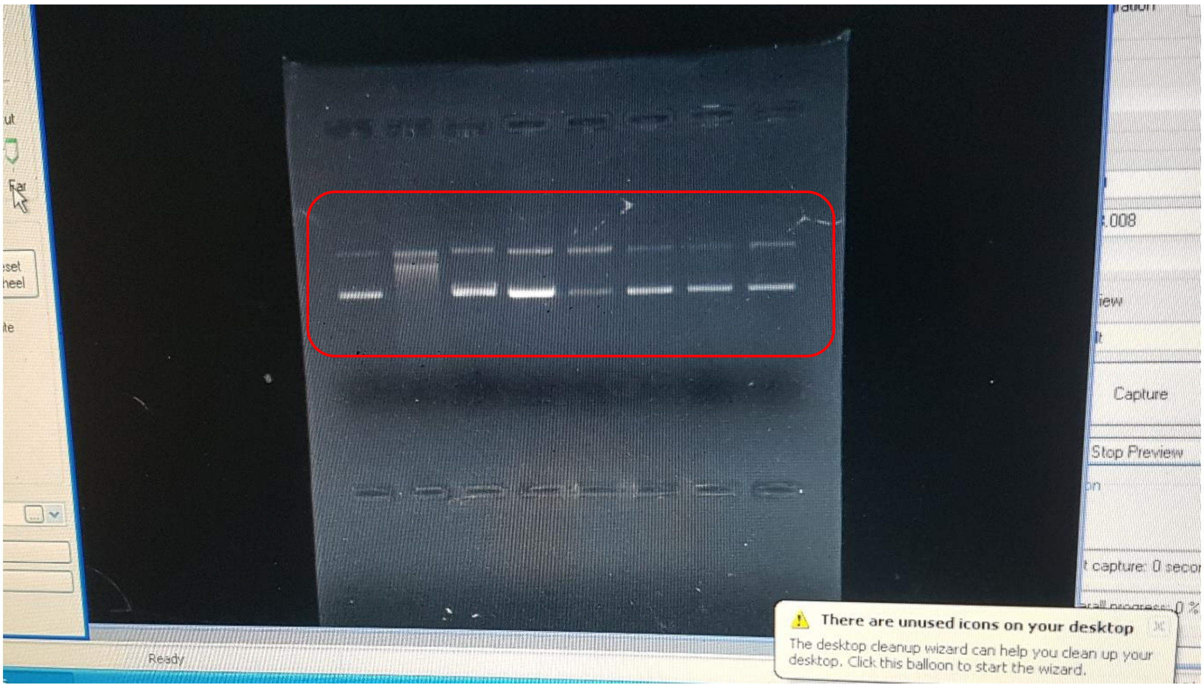

Cropped Figure 2(A)

1 2 3 4 5 6 7 8

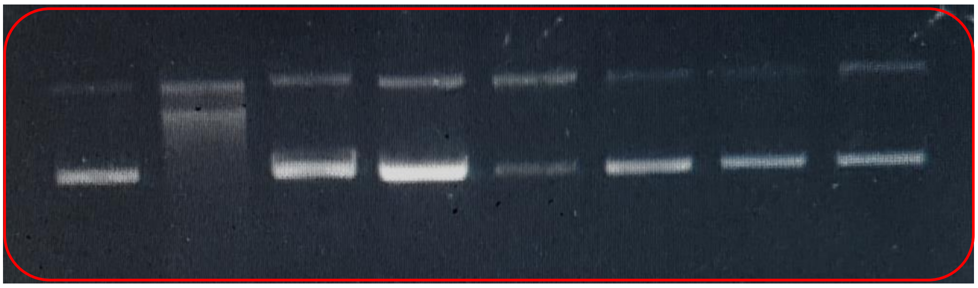

Form II (Nicked Linear)

Form III (Open Circular Relaxed)

Form I (Native Supercoiled)

4. **Supplementary methods sections**
   Section 1: Steps for Gel Image Processing & Gel band quantification

a. **Open the Program:** Open the LabImage 1D software (Kapelan Bio-Imaging, Leipzig, Germany).

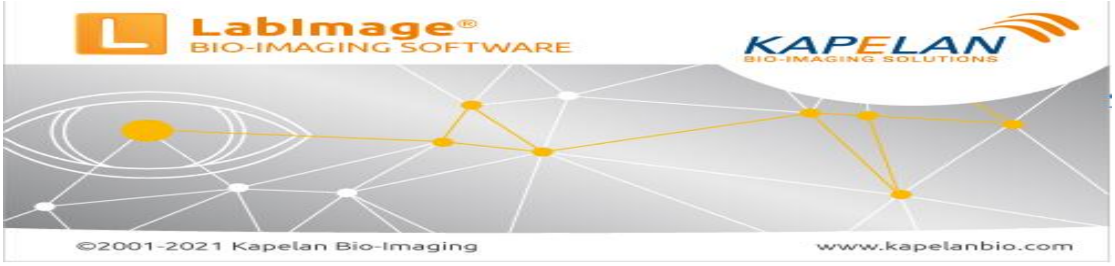

b. **Open Image:** To open the raw image, click the ‘New’ tab and create new project. Open image to be processed in ‘File’ menu.

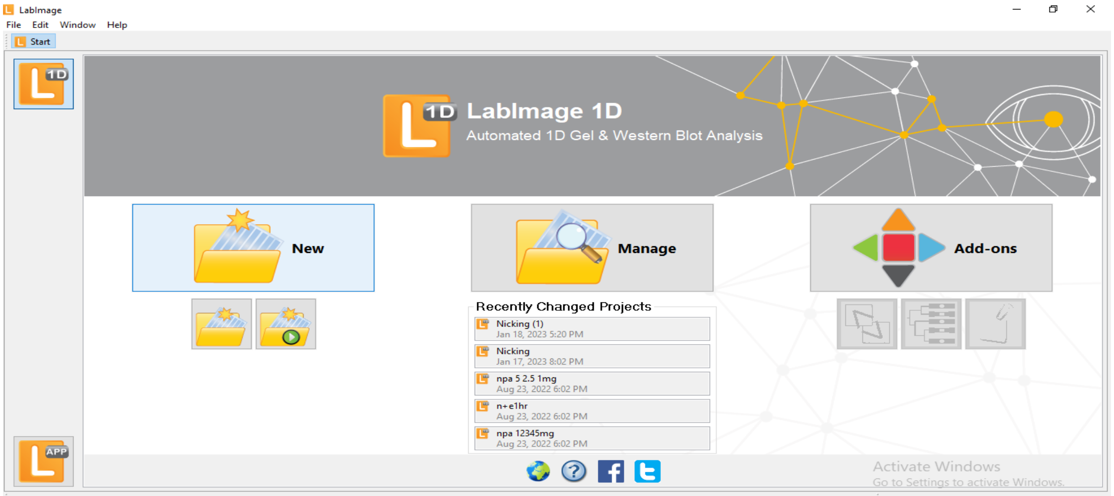

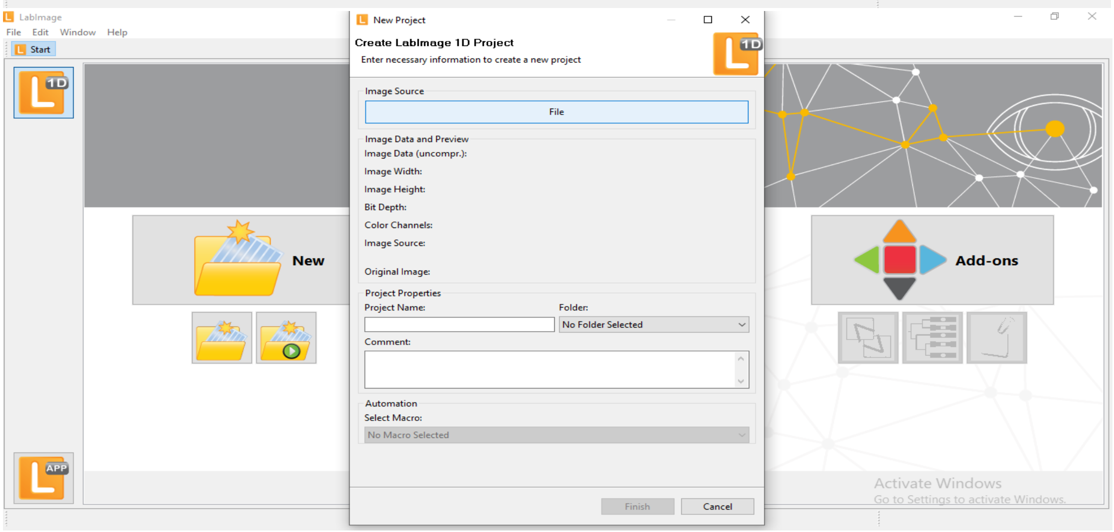

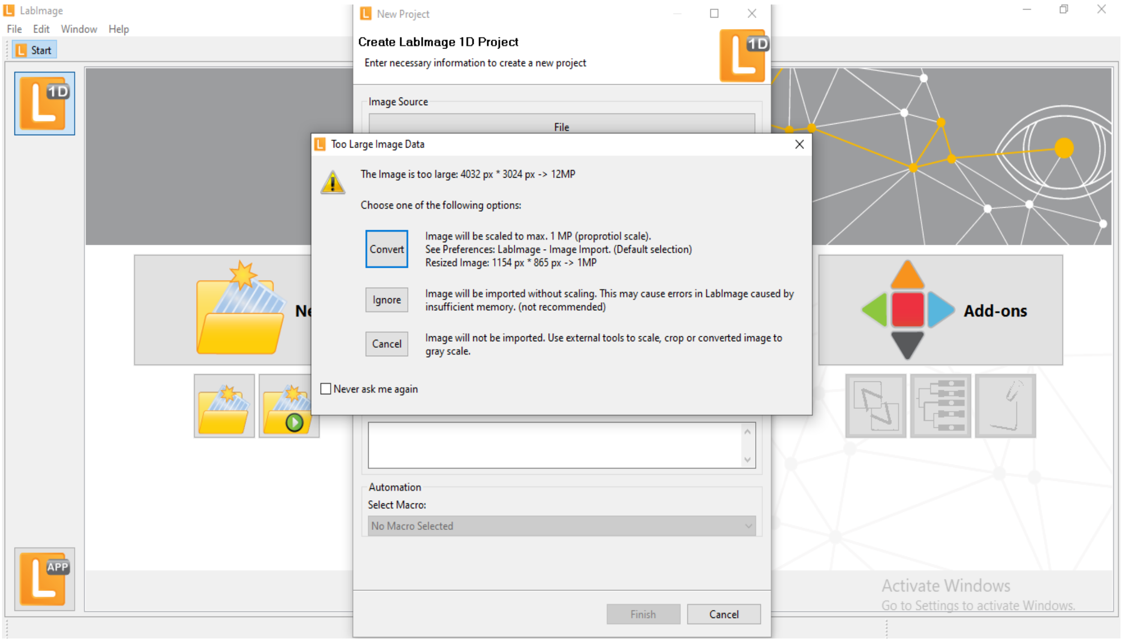

c. **Prepare Image:** Go to Preprocessing menu>Prepare image.

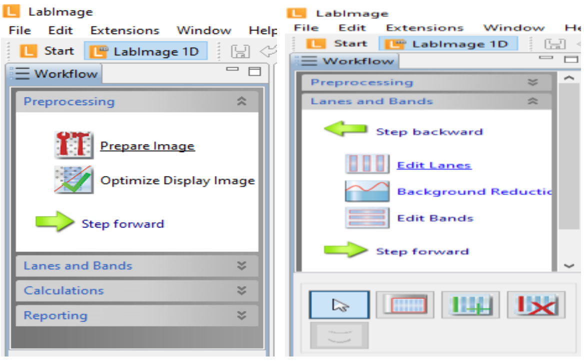

d. Lane & Band detection

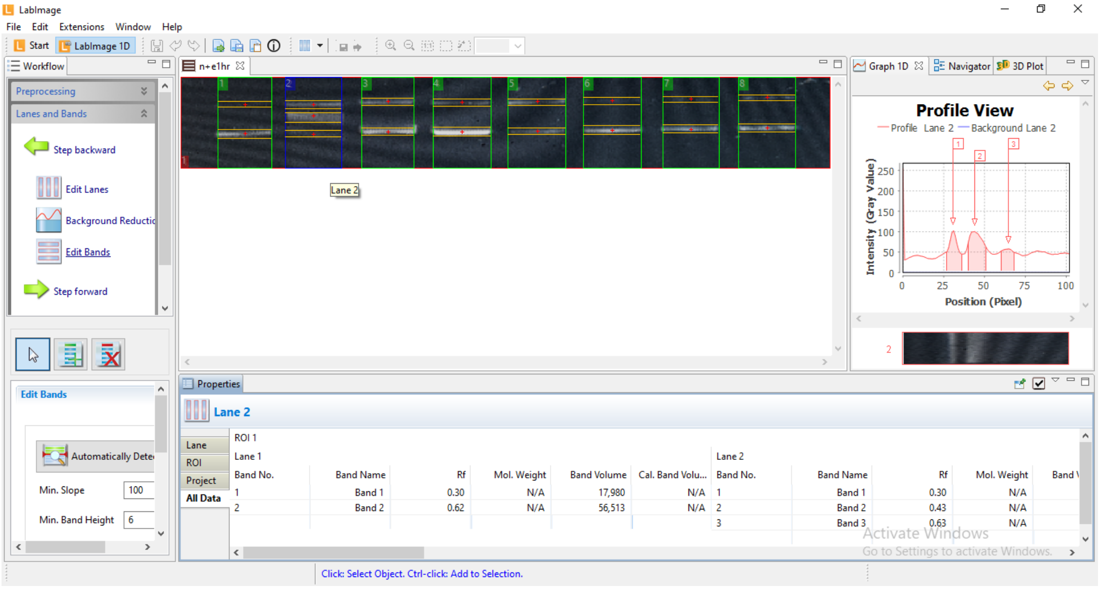

The above steps were followed to quantify the gel images in the manuscript.

e. Proceed similarly with all the bands for each lane.

f. Take a screenshot of 1D graph profile view and save image for each lane in ‘TIF’ file format. Also copy and paste the band volume in Microsoft Excel.

g. Estimate the relative percentage of each DNA band for each lane according to the following equation:

$$Relative\ \%\ \ DNA\ bands = \frac{Actual\ volume\ of\ \ DNA\ band}{Total\ volume\ of\ all\ bands\ in\ lane} * 100$$
